# Supplementary material for: Iterative design of training data to control intricate enzymatic reaction networks
Source: Nat Commun. 2024 Feb 21;15:1602. doi: 10.1038/s41467-024-45886-9 (PMC10881569; doi:10.1038/s41467-024-45886-9)
Supplement: Supplementary file 1 — Supplementary Information [file 41467_2024_45886_MOESM1_ESM.pdf]

Supplementary Information

Iterative Design of Training Data to Control Intricate Enzymatic Reaction Networks

Bob van Sluijs[1], Tao Zhou[1]*, Britta Helwig[1], Mathieu Baltussen[1], Frank H. T. Nelissen[1], Hans A. Heus[1], and Wilhelm T. S. Huck[1] *

[1] Institute for Molecules and Materials, Radboud University, 6525 AJ Nijmegen, The Netherlands.

Corresponding Author: tao.zhou@ru.nl; w.huck@science.ru.nl

Contents

1. Software 4
1.1 Software and background 4
1.2 Defining a model 7
1.3 Defining an observations and simulating models 8
1.4 Training models and OED with a hybrid agent/evolutionary algorithm 9
1.5 Defining a cost function to train the model 11
1.6 Defining an optimal experimental design problem 11
1.7 Summarizing the software with a practical example 13
2. Model(s) of enzymatic reaction network in flow 15
2.1 Coarse graining the model of the ERN 16
2.1.1 Training models with alternative rate laws 16
2.1.2 Testing models with alternative rate laws 18
2.1.3 Allosteric interactions do not significantly affect product formation fluxes 18
2.1.4 Further coarse graining the model, comparing the minimal form of the generalized rate law and BiBi Random equilibrium rate law 19
2.1.5 Demonstrating the limit of the coarse graining process. 21
2.2 A note on parameter estimation methods 23
2.3 Effect of Different Flow Rates on Product Formation 23
3. Experimental Data 25
3.1 Ion Pair HPLC Data 26
3.2 Flow Experiment Data 27
3.3 Output control experiments 31
4. Enzyme Purification and Validation of Enzyme Activity 32
4.1 Enzymes: Source and Availability 32
4.2 Cloning, expression and purification 33
4.2.1 UMPK 33
4.2.2 GMPK 34
4.3 Enzyme immobilization 35
4.3.1 Empty hydrogel beads preparation 35
4.3.2 Immobilization procedure of enzymes on empty hydrogel beads individually 35
4.4 Characterization of Enzyme-beads 36
4.4.1 PK beads characterization 36
4.4.2 AK beads characterization 37
4.4.3 APRT beads characterization 38
4.4.4 UPRT beads characterization 39

4.4.5 UMPK beads characterization 40
4.4.6 GMPK beads characterization 41
4.4.7 Enzyme beads stability characterization 43
5. Supplementary References 44

1. Software

1.1 Software and background

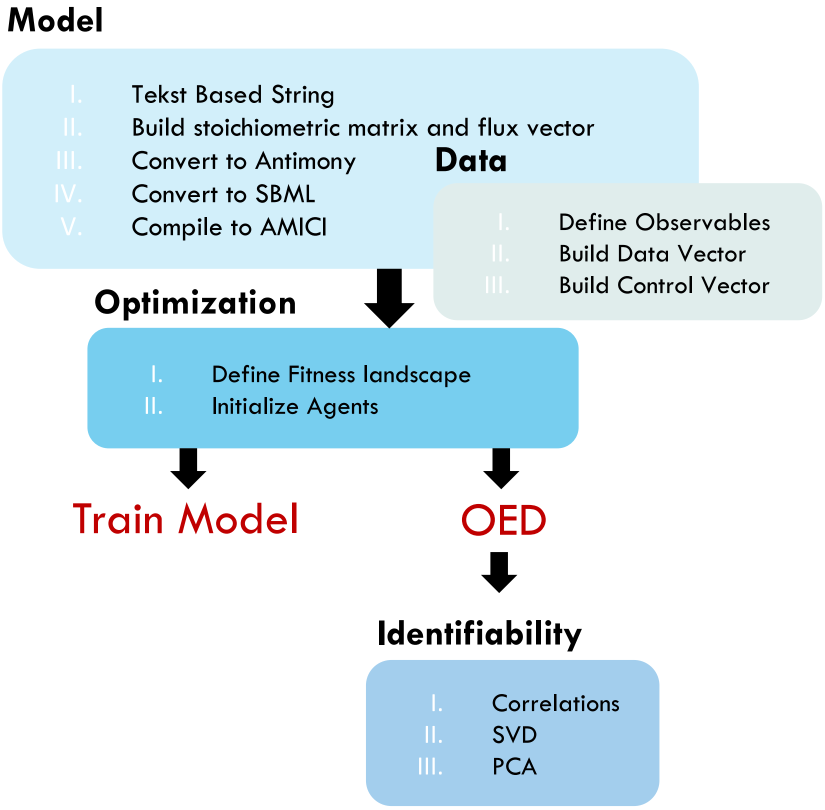

Schema 1. Overview of software, we build and compile models to be optimized.

The software is split in different sections; the first section includes a generalized approach to model notation and compilation, the second section includes a hybrid particle optimization/genetic optimization algorithm, including a module used for optimal experimental design (OED), evolving a set of control parameters of any model to produce maximally informative training data and a module that fits any set of models to a database of experiments. The final section analyses the outcome of the OED process. These tools share the same plug and play codebase so non-expert users can use them. This overview is meant to demonstrate the generalizability of the software to tackle any optimal experimental design problem.

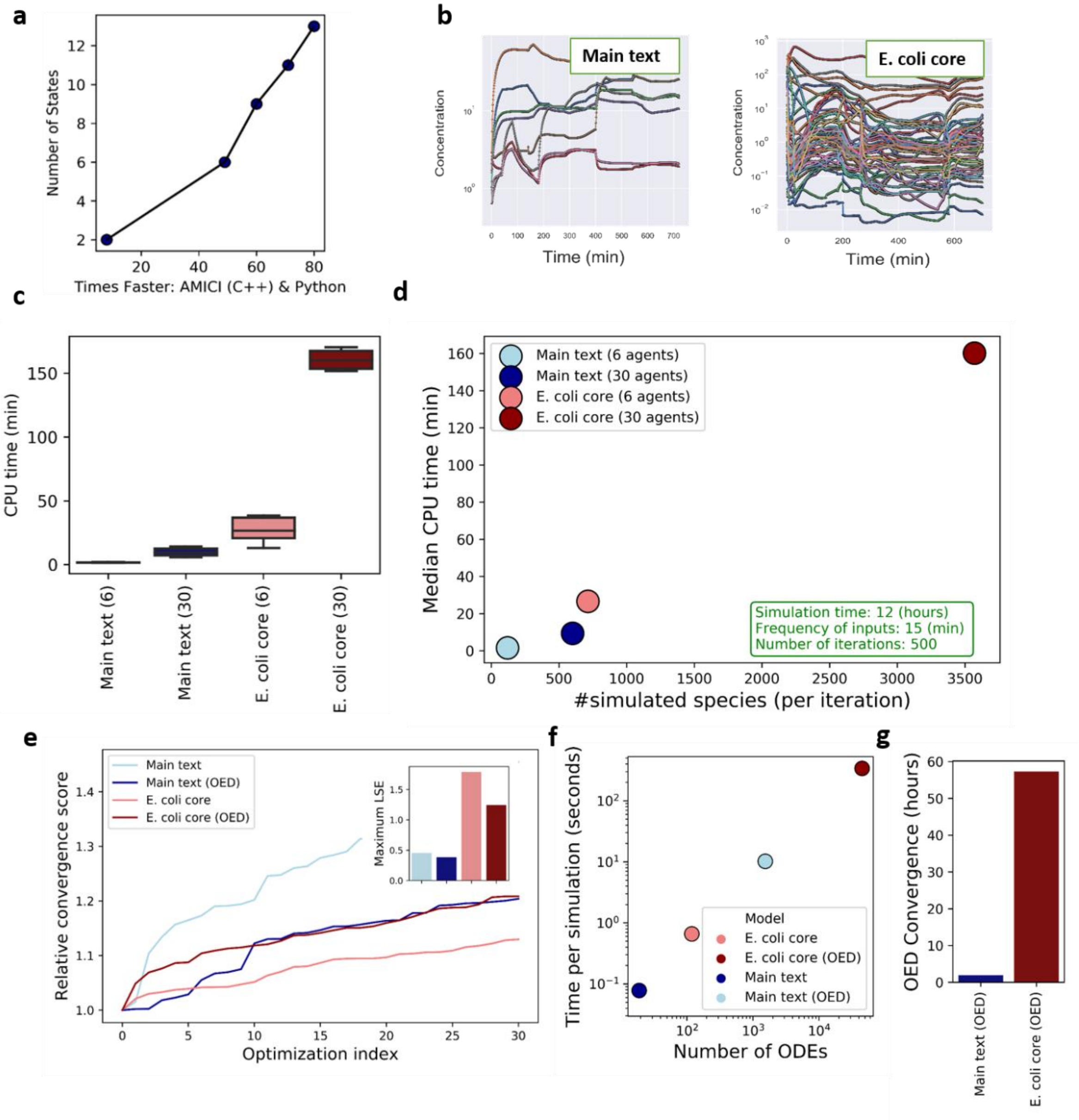

Fig. S1. Shows computational speeds for different aspects of software presented in main text. a) Shows the difference between an ODE model solved in python and AMICI for the number of species the model simulates. b) Shows the time course data (12 hours) of a randomized pulse experiment for both the model presented in the main text (19 states simulated in this version) and the E. coli core model (119 states simulated). The input flow rates are altered every 15 minutes. c) Shows the convergence time for each model trained with either 6 agents or 30 agents (N = 20, the box itself shows the quartiles where middle boxes represent 50%, with a line showing the median value. The whiskers of the box show the highest and lowest values). d) Breaks down c and shows the median CPU given the number of simulated species per iteration i.e., solving a model 6 times means implies it solved 114 ODEs for the first model and 714 solved ODEs for the E. coli core model. e) Shows the relative convergence scores of these optimizations (log normalized summed least squares error), the inset shows the absolute convergence values of the worst fit. f) Shows the average simulation time of each model given the number of ODEs that need to be solved, for the OED version of the models i.e., including sensitivity equations this takes a lot longer. g) Shows the time it takes to optimize a flow experiment for each model utilizing a single core.

The goal of the model module is to map an ODE model (written out in a human-interpretable format) to different open-source packages, specifically compilers that compile the model to a non-interpreted coding language like C++. This is highlighted by Figure S1a where a system of 13 ODEs solves 80 times faster in AMICI than python. To test the scalability of the entire active learning approach software, we opted to test its speed applied to two models (the tests were run on a single Intel Xeon E5-1660 v4 @ 3.2 GHz core 2017). Specifically, the network/model featured in the main text and the *in vivo* metabolic core *E. coli* model from literature (Khodayari et al. 2014) placed in an *in vitro*, context.

Computational speed: models

We test the speed of both the training of the model and the optimal experimental design for 2 models:

- Main text model (free enzyme):
  - **20** reactions
  - **19** simulated species (all metabolites observed)
  - **1349** sensitivity ODEs
- *E. coli* core metabolism model (free enzyme):
  - **100** reactions (modified and removed transport and degradation from model)
  - **119** simulated species (all metabolites observed)
  - **44982** sensitivity ODEs

We maintain reactor set-up akin to the one used in the main text yet utilize 12 syringes (maximum without modification) that flow both enzymes and substrates into the reactor with different inflow rates every 15 minutes (making the problem stiffer). The summed inflow rate of these syringes determines the total outflow rate of the species within the reactor. For the purposes of this test, we include all species, enzymes and metabolites (all observed) in the stock solution of the syringe, the inflow rates of individual syringes can change every 15 minutes. This means we do not allow the system to go towards steady state during the experiment, making the problem stiffer, thus increasing the computational load. For the *E. coli* model, the enzymes are divided over the first 10 syringes, the energy carrying molecules (*Glucose, ATP, NADPH* etc.), are combined in a single syringe, all other substrates and products in another, single syringe. For the original model each enzyme gets its own syringe.

Both models are initialized with random set of parameters and we build an *in silico* dataset by simulating a pulse experiment for 12 hours. We applied a series of randomized time dependent pulses to both networks (15 minutes intervals). This results in a simulated time course dataset for both models shown in Fig. S1b (left = main text model) and S1c (right = *E. coli* core model). The xml files for the model can be found at github.com/huckgroup/OED.

Computational speed: training a model

When we train the model, we change a single hyper parameter for each, the number of agents that move across the fitness landscape (i.e., parameter mutations that are simulated each iteration). Figure S1c shows the distribution of convergence times for each model. Summarized, a single CPU:

- ~**80 seconds** to converge (main text model trained by 6 agents).
- ~**7.5 minutes** to converge (main text model trained by 30 agents).
- ~**25 minutes** to converge (*E. coli* core model trained by 6 agents)
- ~ **155 minutes** (*E. coli* core model trained by 30 agents)

In Figure S1d it breaks this down in median CPU time per the number of total ODEs per iteration that had to be solved for each model. In Figure S1e we show the relative convergence least squared error score for

each model/optimization combination with the absolute error in the inset. This shows that a small number of agents leads to identical fits for a small model but, for the *E. coli* core model, more agents per iteration improve the fit (the translated *in vivo* model is significantly stiffer than the model from the main text).

Computational speed: optimal experimental design of control inputs

For optimal experimental design of the control inputs the sensitivity equations need to be solved to the sensitivity matrix. This equates to unique ODEs for every parameter and state combination. The model from the main text has 19 ODEs plus 1558 sensitivity ODEs, the *E. coli* core model has 119 ODEs plus 44982 sensitivity ODEs. In a practical example we will never have this many observables and we do not need the sensitivity of all parameters e.g., inflow rates, to all states, which would drastically reduce the size of the sensitivity matrix. However, we assumed this was the case to test the OED software. We optimized the control inputs of 12 syringes for both models with 6 agents across 100 iterations. Figure S1g shows it takes less than 2 hours for the smaller model to converge on a single CPU whereas the larger model takes 58 hours. When we test the average simulation times of a single model this breaks down according to Figure S1f.

- Main text model: **0.078** seconds
- Main text model + sensitivity equations: **10.2 seconds**
- *E. coli* core model: **0.66** seconds
- *E. coli* core model + sensitivity equations: **339 seconds**

Summarized these numbers give a rough indication of the computational times involved. We note that the current *E. coli* core model exceeds the scope of currently feasible *in vitro* applications yet still remains computationally solvable within the timeframe of a single experiment (even on a single core).

1.2 Defining a model

We define a model, including;

I. Model states
II. Observed states
III. Parameter Prior
IV. Lower and upper bound for each parameter
V. Control parameters

The model itself can be defined by writing out the equations as strings. This template is subsequently processed in a model module. First, the ODEs are transformed reformatted into a matrix, after which we obtain a stoichiometric matrix and a flux vector. Derived from the ODEs original reaction equations this would be defined as

$$\sum_{i=1}^{N} \mathrm{R_{ij}X_i} \xrightarrow{\mathrm{k_j}} \sum_{i=1}^{M} \mathrm{P_{ij}X_i} \tag{1}$$

Were $j \epsilon [1, N]$. Thus **R** and **P** are N x M matrices representing the number of products in a reaction, respectively, and k is a vector of N reaction rate. Note that any type of kinetic approximation can be applied (Michealis Menten or Hill type kinetics). This system is subsequently translated to a set of ODEs in matrix format according to

$$\frac{d\mathrm{X}}{dt} = Sv(\mathrm{X}) \tag{2}$$

Where $\boldsymbol{S} \triangleq (\boldsymbol{P}-\boldsymbol{R})^T$ is the stoichiometry of the system and $v(X)$ the kinetic equations in the flux vector. This stoichiometry matrix and flux vector are subsequently used to create an *Antimony*[2] file (human readable version of SBML, standard biological markup language) using the publicly available *Tellurium* toolbox[3]. Antimony makes use of reaction equations and fluxes in their standardized notation of models, by creating a flux vector and stoichiometric matrix it becomes easier to translate our original ODEs from equations to antimony. Next the antimony file is converted to an SBML[21] file using *e.g.*, which in turn can be compiled to C++ using *AMICI*[1], all intermediate versions of this model are stored in the model module, the general process is shown in schema 1. Finally, the module already defines the parameter space by building a (uniform)logarithmically spaced vector V with n the size of the vector (1000). The individual parameters $V_i = 10^{\theta_i}$ where θ_i is sampled from a uniformly distributed vector $\boldsymbol{\theta}$ defined by:

$$\boldsymbol{\theta} = \{\mathrm{r}: \mathrm{r}\ \exists \mathrm{i} \in \mathbb{N} \text{ such that } \mathrm{r} = \log_{10}(\alpha) + \frac{\log_{10}(\beta)}{\mathrm{n}} i, \text{and } \mathrm{r} \in \left[\log_{10}(\alpha), \log_{10}(\beta)\right)\}\} \tag{3}$$

where α and β, the previously defined upper and lower bound of a parameter. This means that each possible value of a parameter has a corresponding integer index.

1.3 Defining an observations and simulating models

To integrate experimental data, experiments -either gathered from the *in vitro* set up, or simulated *in silico*- are defined as a separate module. The data is parsed into data vectors, these vectors are (optionally) interpolated using a Savitz-Golay filter[5]. The size of the interpolation window and the power law terms are defined by the user and can be tuned to account for data sparsity (as to not introduce mistakes during the interpolation process). The conditions of the experiments are passed along as a separate vector, this includes both initial conditions and time dependent inputs. The latter are defined as separate arrays noting the start and end time with a dictionary vector containing the control parameters:

$$\begin{bmatrix} P_1 \\ \vdots \\ P_n \end{bmatrix}_{(t_{start_1} \to t_{end_1})}$$

$$\vdots$$

$$\begin{bmatrix} P_1 \\ \vdots \\ P_n \end{bmatrix}_{(t_{start_a} \to t_{end_b})}$$

Where each time window $t_{start_a} \to t_{end_b}$ for a control input is defined by the time window over which that input is applied and the values P of the control parameters within this window. Finally, if no experimental data is available, the option exists to generate the data *in silico* by adding noise to the simulated outcomes from the model module and redefining it as a measured vector. Any model can be combined with any measurement object to form a dataset (see Fig S3 & S4).

To actually solve the ODEs, we build a solver module that can simulate the ODEs in different formats, notably this solver needs to be capable of simulating the time dependent inputs. The model solver takes the model module and the measurement object and simulates the system. If it cannot an AMICI object, Tellurium will be used, if that is lacking Scipy's integrate (LSODA) will be used, if no measurement object is provided the control parameters will default to values defined in (2) or a vector defined manually.

1.4 Training models and OED with a hybrid agent/evolutionary algorithm

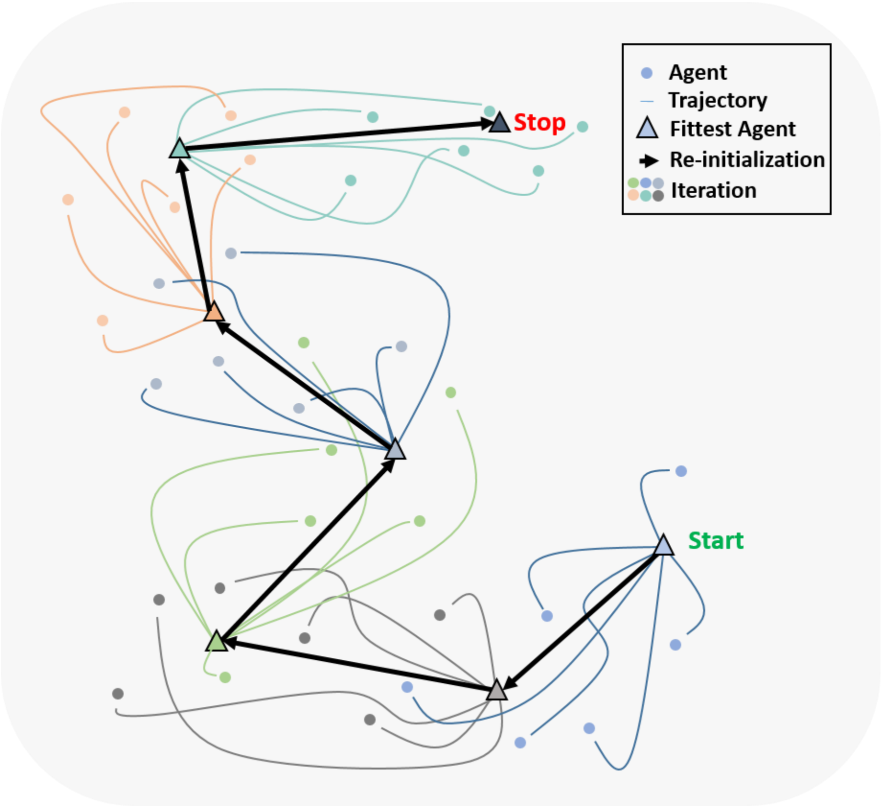

Fig. S2. Abstraction of the evolutionary/particle optimization algorithm, the start location of the agents is chosen by selecting the fittest position on the fitness landscape after a Monte Carlo type search (sample 1000 parameter sets). The agents are subsequently mutated shifting their parameter values by perturbing it locally or by applying global perturbations, a new value to a parameter at random. In this process, the agents (circle) move independent from one another, and the agents only move if the mutation improves fitness. After a set number of iterations (default = 20, each colour represents an iteration) the agents (dots) communicate, the unfit agents coalesce around the fittest agents (triangle). This process is repeated until the agents converge to a local optimum.

We developed a hybrid particle/evolutionary optimization algorithm capable of optimizing a model or set of models towards any objective function. The module is split into 2 parts;

I. Fitness landscape
II. Agents

Both can be summarized abstractly: in the fitness landscape module we define the scope of the optimization problem, including the objective function, the number of agents that will move across the fitness landscape, the selection criteria of agents and the manner in which they move across said landscape as a collective. The agent contains all the relevant information pertaining to its own specific location on the fitness landscape, its past trajectory across the fitness landscape, and the manner in which it moves across the landscape[6,7]. Numerous meta-heuristic algorithms have been published and applied to standardized benchmark functions which are reportedly good at searching complex fitness landscapes[8]. These benchmarks are in effect a fitness function which shapes the fitness landscape (e.g. imagine a value with peaks and troughs, where the peaks represent an optimal solution, the troughs a bad solution). The algorithm effectively represents the search pattern the agents utilize to navigate this landscape. Since the late 90s a

score of these abstract algorithms have been published based on ever more 'inventive'[5,] search pattern equivalents from 'nature'[9,10,11]. Thus, algorithms appeared; from mimicking the bubble net hunting technique of whales[12] to mapping and mimicking the behaviour of; ant colonies[14], Dolphins[15], glow-worms[16], elephants[17], migrating birds[18], imperial conquest[19] even a group therapy session[20].

Importantly, the fitness function and its accompanying fitness landscape is the ultimate arbiter of these meta-heuristic algorithms and favours one over another interchangeably depending on its current definition (genetic algorithms remain well suited to all types of problems[9,25]). However, we note that within this chorus of algorithms some features do matter, namely, freedom for a subset of agents to err and explore dead ends, and exchange of information between these agents and those that are moving towards an optimum[8]. **This especially counts for very non convex problems e.g., training a model of a non-linear system to multiple experiments where that network is being perturbed constantly.**

Figure S3 encapsulates the core of our optimization algorithm. First the frame needed to be capable of handling multiple models and experiments simultaneously (including time dependent inputs). With respect to the search pattern, we attempted to keep the algorithm simple whilst maintaining the freedom needed to fit multiple models and experiments simultaneously. We initiate a number of agents on the fitness landscape based on an initial Monte Carlo search, sampling parameter sets (default = 1000), simulating these and selecting the fittest sets as initial starting points for the optimization. Each iteration the agents (default = 10) are mutated (a fraction 0.25, is recombined with other agents), if the mutation leads to better convergence with the data (or improved score), they move across the landscape, after a set number of iterations (default = 25), the agents are collected around the fittest agent in a recombination step, after which they are to move independently again. This process is repeated until the cost function that defines the optimization no longer improves or after a defined number of iterations (default 300).

Practically this subroutine can be applied to any optimization problem using the model module. The model parameters are the mutable objects within the agents. When we optimize a set of control experiments, the control parameters are allowed to mutate. If we are training a model, the -to be estimated- rates are allowed to mutate, each by modifying the parameter index defined in (4). The size of these mutation can either be small (shifting the index by 1 to 3 integers up or down the parameter space) or large (shifting the integer by any number up or down the parameter space). Additionally, the option exists to use the forward sensitivities to determine the direction in which a parameter needs to be mutated to improve the fit. To apply a directed mutation, we calculate the forward sensitivity with respect to the likelihood and where we end up with the following equation

$$\frac{dL(Y:p)}{dp} = \sum_{i=1}^{n}\big(y(t_i) - x(t_i, p)\big) \quad * \quad x_p(t_i, p) \tag{4}$$

Thus if $y(t_i) - x(t_i, p)$ is positive i.e., the model predicts on average lower concentration than the measured data, but the forward sensitivities of a parameter p_i are on average negative, the term on right hand side of the equations becomes negative; lowering the value of parameter p_i will cause the model to converge with the data.

The agents can incorporate any single or set of experiments when training a model, thus any model can be matched with any experiment. This makes it possible to optimize different models to the same dataset simultaneously or different models with shared parameters (e.g., same structure different context) to different experiments (Schema 2 & Fig S4). When agents mutate a parameter, this mutation is superimposed onto all the models associated with the experiments. This is illustrated in schema 2.

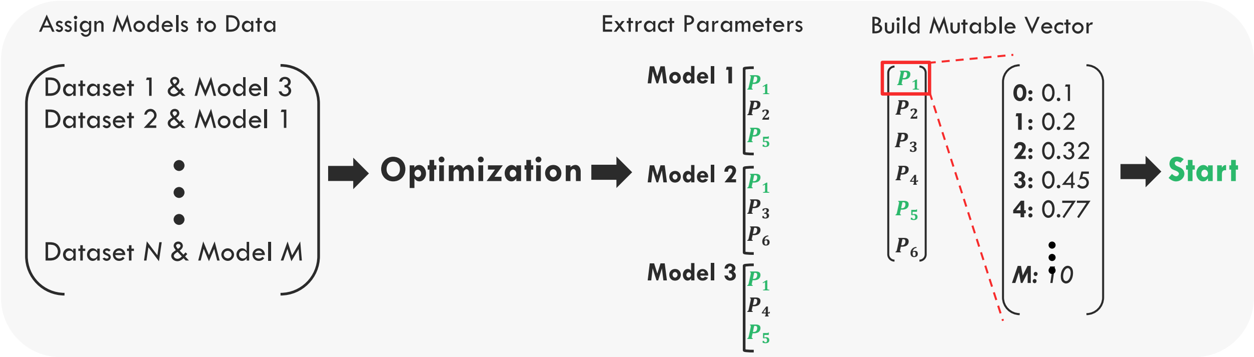

Schema 2. Overview, we assign models to specific datasets, in the optimization module, these models are 'combined' in a single overarching model by combining the parameters in a single vector, if two models have the same parameter, they are mutated (simulated separately).

1.5 Defining a cost function to train the model

Fitting the model is a non-trivial problem since the experimental dataset is complex, consisting of multiple experiments where the state space is constantly perturbed by alternating values for the control parameters.

$$C = \sum_{k=1}^{i} \sum_{l=1}^{o} \frac{\sum\left(\left(N_{k,l}\right) - \left(M_{k,l}\right)\right)^2}{\left(\frac{\sum_{j=1}^{n} M_{k,l}}{n}\right)} * D \tag{5}$$

Where N is the experiment and M the corresponding simulation time course data vector, I the index of the experiment, o the observable and n the timepoint, D is the relative deviation (factor) between observed mean in the data and the simulated mean over time.

1.6 Defining an optimal experimental design problem

Raue *et al.* defines 3 types of identifiability[20];

- identifiable
- practically unidentifiable
- structurally unidentifiable

If a parameter is identifiable, the data is informative enough, or the model simple enough to learn the true rate, the sharpness of the parameter likelihood is often defined by the measurement noise in this case. If a parameter is practically unidentifiable the measurement is simply too noisy to find a true rate. If the parameter is structurally unidentifiable, parameters can directly compensate for one another and any combination of parameter values will map onto the production fluxes within a biochemical network. This mathematical reality presents us with a set of choices, we can opt to keep the model as simple as possible, this allows us to identify the rates, but the predictive power will be limited if the mechanistic detail is not sufficient to map onto the production fluxes. However, expanding the model by adding parameters, would come at the cost of the structural identifiability, introducing more uncertainty. To manage this, the training data used to fit a model needs to be sufficiently complex, such that the parameters, at a minimum, map onto the right conversion flux when conditions change (whereby the model has some predictive power).

Fortunately, ODEs have interesting properties, specifically it is possible to quantify the collinearity between parameters for any individual or set of experiments and a given dataset, a feature that is leveraged in the field of optimal experimental design (OED). Most optimal design problems revolve the fisher information matrix (FIM)[20,21,22,26], this metric is defined as the second derivate of the parameter likelihood $L(Y\colon p)$,

geometrically it computes the curvature around the maximum likelihood estimate (sharp or flat). This matrix can be computed from the forward sensitivities of the ODEs, by differentiating the observables with respect to the parameters according to [1,7]

$$\frac{d}{dt}\frac{\partial x}{\partial p_i} = \frac{\partial f(x,u,p)}{\partial x}\frac{\partial x}{\partial p_i} + \frac{\partial f(x,u,p)}{\partial p_i} \quad for\ i = 1, \dots, N_p \tag{6}$$

Where $\frac{\partial f(x,u,p)}{\partial x}$ is the Jacobian, $\frac{\partial x}{\partial p_i}$ the sensitivity coefficients and $\frac{\partial f(x,u,p)}{\partial p_i}$ the parameter sensitivities. Note, we end up with an additional $N_p * N_x$ number of sensitivity ODEs. By differentiating the original ODEs with respect to the parameters we effectively map how much each parameter contributes to the rate of change in the observed species. The sensitivity matrix can subsequently can be obtained by stacking these sensitivities towards different observables at different times according to:

$$\boldsymbol{S} = \begin{bmatrix} \frac{\partial y_{j1}}{\partial p_1}(t_1) & \cdots & \frac{\partial y_{j1}}{\partial p_{N_p}}(t_1) \\ \vdots & & \vdots \\ \frac{\partial y_{j1}}{\partial p_1}(t_{N_t}) & \cdots & \frac{\partial y_{j1}}{\partial p_{N_p}}(t_{N_t}) \\ \frac{\partial y_{j2}}{\partial p_1}(t_1) & \cdots & \frac{\partial y_{j2}}{\partial p_{N_p}}(t_1) \\ \vdots & & \vdots \\ \frac{\partial y_{j2}}{\partial p_1}(t_{N_t}) & \cdots & \frac{\partial y_{j2}}{\partial p_{N_p}}(t_{N_t}) \end{bmatrix}$$

Where y is are the subset of observable states x, p the parameter and t the time point of the observation up to t_{N_t} and j the index corresponding **to individual experiments**. Thus, each column is a parameter, each row a time point and within a single column we can stack the sensitivity of that specific parameter towards different observables observed in different experiments. With this, The FIM is subsequently computed according to

$$\boldsymbol{F} = \boldsymbol{S}^T\boldsymbol{S} \tag{7}$$

To score the amount of information about the parameters is present in F we can make use of different optimality criteria. D-optimality is calculated by taking the determinant $\max_D(F)$[22], it effectively calculates the volume of the parameters space wherein optimal convergence for a given dataset can be found. In contrast, the E-optimality metric can quantify the length of the diagonal in the parameter cloud. This approach is described by Brun *et al* and Gabor *et al.*, we apply a single value decomposition operator to the FIM according to $SVD(\frac{\boldsymbol{F}}{||\boldsymbol{F}||})$[21,23]. The collinearity index (CI) corresponding to the length of the diagonal through the parameter cloud can subsequently be calculated according to $\frac{1}{\sqrt{\min(\lambda)}}$ where $\min(\lambda)$ is the smallest eigenvalue (the single value decomposition is a mathematical operation that breaks a matrix down into 3 constituent parts)[1,4].With this, we can compare to what extend parameter pairs or groups can compensate for one another, thus we can compute which experiments are best suited to identify a specific parameter.

Note, that the structure of the fisher information matrix highlights the need for reasonable priors. F is built using the derivatives of the parameters we are trying to estimate. Thus, if the initial parameter estimates are very far of their true value the computed optimality criterion, it is not representative of the actual system,

causing either an underestimation or overestimation of the amount of information present within the experiment[24]. Nonetheless, this matrix can be leveraged in different contexts, from finding the most informative time points to take measurements to mapping complex non-linear systems by breaking collinearities, learning more about the larger 'kinetic landscape' in the process.

1.7 Summarizing the software with a practical example

In S3 and S4 we show a practical example of the code to utilize al the modules describe in 1.1-1.5, the user can define the model manually in a separate python file (S1). Note that the naming in the models, its states and parameters needs to be consistent, similarly when you define the model make sure that any subtraction or addition inside a **fluxterm v in (2) is marked by straight lines '|+|' and |-|,** anything between two unmarked signs will be considered a fluxterm and mapped to equation (6).

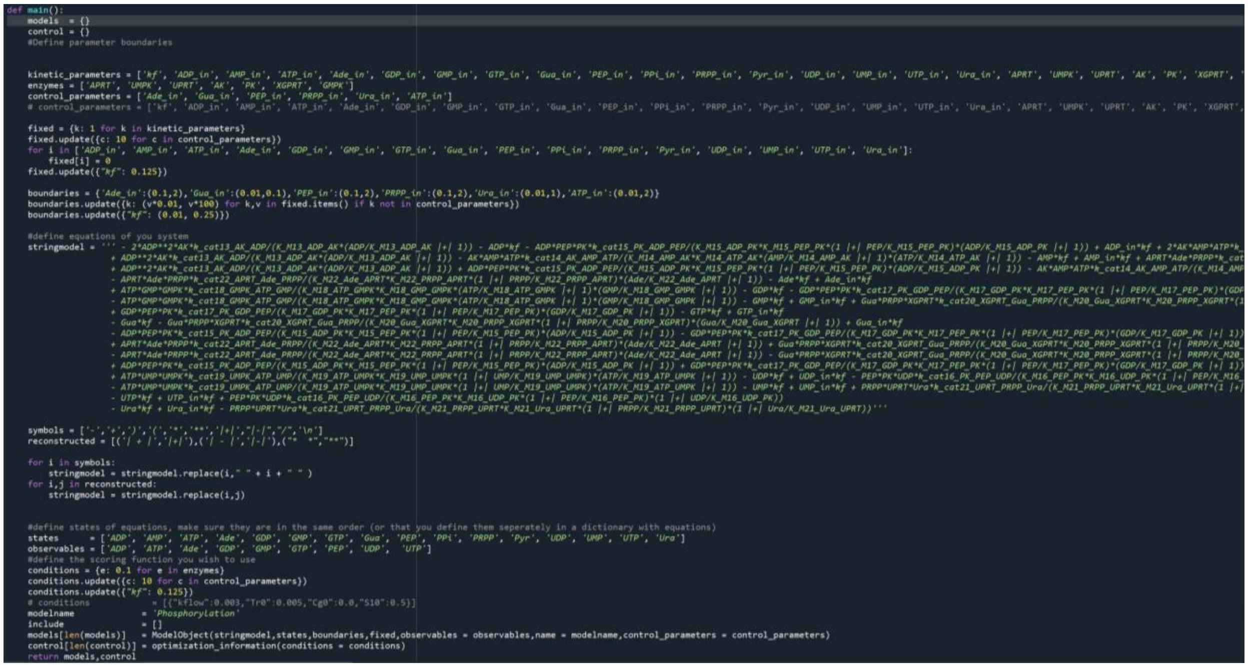

Fig. S3. The input file that the software uses. One simply defines any ODE model and includes which symbols belong to which category (state variable, parameter, control parameter).

This model file can subsequently be called from the main file shown in S4. In this file we import the modules needed to compile a model, define the experiments (either real or simulated) and combine these to optimize any experiment and train any model or set of models to any number of experiments. Summarized figure S4 shows us importing the model module shown in S3, we generate a number of experiments, we combine both the dataset and its corresponding model in a simple dictionary. This dictionary is the input for the model training model. There is a full description of the software available at github.com/huck/OED. We include model building software that can take a list of any enzymatic reaction network defined by the: enzyme, its reversibility, the kinetic rate law, its substrates, its products, its inhibitors and activators.

'PK',	True,	'GH',	['ADP','PEP'],	['Pyruvate','ATP'],[],[]
'UMPK',	True,	'GH',	['UMP','ATP'],	['UDP','ADP'],[],[]
'GMPK',	True,	'GH',	['GMP','ATP'],	['GDP','ADP'],[],[]
'AK',	True,	'GH',	['AMP','ATP'],	['ADP','ADP'],[],[]
'APRT',	False,	'GH',	['PRPP','Adenine'],	['AMP'],[],[]
'UPRT',	False,	'GH',	['PRPP','Uracil'],	['UMP'],[],[]

```python
42 """import the model"""
43 import ToyModel as model
44 models,control = model.main()
45 
46 """create th SBML model and Amici models, needed because packacges conflict"""
47 for i in range(len(models)):
48     model = models[i]
49     model.SBMLconversion()
50     model.PytoCompile()
51 
52 """import modules for experiment generation and identifiability analysis"""
53 from Measurements import GenerateExperiments
54 from IdentifiabilityAnalysisModelSets import IdentifiabilityAnalysis
55 
56 """name of folder where identifiability of experiment will be stored"""
57 name = model.name + 'First Test'
58 
59 """create a 'fake' measurement objects in the GenerateExperiment class
60 (you can do batch, random pulse etc. just check measurement folder)"""
61 m = GenerateExperiments(model)
62 measurement_1 = m.return_random_pulse_measurement(store = True,name = name,time = (0,100))
63 measurement_2 = m.return_random_pulse_measurement(store = True,name = name,time = (0,100))
64 #...
65 #...
66 #...
67 
68 """its a dict {0:Measurement object}, lets combine 2  experiments together and
69 give them to the identifiabilitiy analysis (i.e. it combines them and does the idenfiability analysis as if its 1)"""
70 dataset = {}
71 
72 """build a dataset, Note we only have 1 model but
73 the dataset can consists of measurements with multiple models"""
74 dataset[len(database)] = measurement_1
75 dataset[len(database)] = measurement_2
76 
77 """ANALYSE: Collinearity index, correlation and PCA"""
78 EXP = IdentifiabilityAnalysis(dataset)
79 EXP.Correlation(name = name)
80 EXP.PCA(name = name)
81 EXP.SVD(name = name)
82 
83 """FIT: fit the measurements and retrieve parameter estimates"""
84  BoxplotOptimization(database,
85                      optimization_number = 100,
86                      ,generations = 10,
87                      ,agents = 10,
88                      ,storedata = '[LOCATION]',
89                      startsamples = 1000)
```

Fig. S4. Import the model (47-50), name the model (57), build a dataset of models (61-63), in this example we do not import real experiments but simulate them by randomly changing the control inputs. (74-75) assemble the individual experiments in the dictionary, and finally do some analyses e.g., quantify identifiability (single value decomposition), correlations between parameter sensitivities, PCA analysis.

2. Model(s) of enzymatic reaction network in flow

The coarse-grained model used in the main text assumes reversible generalized bi-substrate kinetics for the enzyme which convert input substrate to a final product according to:

$$V = \frac{[S1][S2][Enzyme\ ]k_{cat}}{Km_1 Km_2\left(1 + \frac{[S1]}{Km_1}\right)\left(1 + \frac{[S2]}{Km_2}\right)} \tag{8}$$

Where [S1] and [S2] are the substrate concentrations, [enzyme] the concentration of beads multiplied by their activity (S25-S36), k_{cat} the catalysis rate of the enzymes. The rate law is very similar to a reversible BiBi ping pong (rapid equilibrium) as described in Cook and Cleland's "Enzyme kinetics and mechanics" with the exception of the Km_1, in this form is identical to assuming: $K_a = K_{ai}$ (see equation 12, or page 83-84 of Cook and Cleland, 2007)[27]

First noted in 2006 in 'a general rate law for systems biology' they derive a generalized hill equation, they demonstrate it maps onto conversion fluxes similar to other rate laws assuming a hill coefficient of 1[30], but it negates the use of an additional parameter Kai. In this context the flux for a reversible reaction subsequently within a microfluidic flow reactor becomes

$$\frac{dX_i}{dt} = V_{Forward} - V_{reverse} + Kf_{in,I}X_{in,stock} - Kf * X_i \tag{9}$$

Where the forward and reverse reactions are noted by V (with different kinetic rates), and the inflow $Kf_{in,I}$ and outflow Kf are proportional to the stock concentration and the concentration in the reactor respectively. For our model we assume that reactions catalysed by *UPRT* and *APRT* are not reversible whereas reactions catalysed by *PK*, *AK*, *UMPK* and *GMPK* are reversible. The inflow rate of input substrates is modelled and accounts for individual syringes connected to the CSTR reactor. Thus, the concentration of the input substrate in a syringe is larger than the final concentration in the reactor (as multiple syringes are connected to the CSTR), and is modelled according to:

$$[X_{ss}] = X_{in,stock}\frac{Kf_{in,I}}{kf} \tag{10}$$

Where $[X_{ss}]$ is the concentration of a single substrate that flows into the reactor at steady state. $Kf_{in,I}$ is the flowrate of a specific substrate/syringe combination that flows into the reactor. $X_{in,stock}$ is the stock concentration of the substrate. Each $Kf_{in,I}$ i.e., substrate/syringe combination contributes to the total flowrate Kf. The total flow rate (flow out of the reactor) that applies to all species is defined as: the sum of all $Kf_{in,I}$.

$$kf = \frac{\sum_{i=1}^{i} Kf_{in,I}}{\text{volume}} \tag{11}$$

We have 6 input syringes for the microfluidic flow set-up. To prevent backflow a minimal inflow of 6.75 uL/hour for each individual syringe is required, the volume of the reactor is 100 uL. Thus, the minimal flow rate in the device is 40.5 uL/hour, for reference, a total flow rate of 100 uL/h translates to a kf parameter of 1/hour i.e. This flow rate is equivalent to the replacement of a single reactor volume. Assuming a

maximum total flow rate of 10 reactor volumes per hour, the controlled substrate concentration range spans > 2 orders of magnitude. To automate the control of the syringes the output of the OED algorithm is translated into a command including the duration of an input and the flow rate of each individual syringe. This text file is subsequently loaded into the pump flow setup interface.

2.1 Coarse graining the model of the ERN

To highlight the coarse graining process of the model (eq. 8-9) in the main text and the identification of potential allosteric interactions we built models where the reaction kinetics of the enzymes are governed by subtly different rate laws as described in the afore mentioned Cook and Cleland "Enzyme Kinetics and Mechanism" (page 84)[26] including:

BiBi ping-pong (rapid equilibrium random)

$$V = \frac{[S1][S2][Enzyme\ ]k_{cat}}{KaiKb + Kb[S1] + Ka[S2] + [S1][S2]} \quad (12)$$

BiBi ping pong (equilibrium ordered)

$$V = \frac{[S1][S2][Enzyme\ ]k_{cat}}{Kai\ Kb + Kb[S1] + [S1][S2]} \quad (13)$$

BiBi Ping Pong

$$V = \frac{[S1][S2][Enzyme\ ]k_{cat}}{Kb[S1] + Ka[S2] + [S1][S2]} \quad (14)$$

The allosteric interactions were subsequently modelled by including an additional term, where the K_{cat} of production flux V is proportional to the concentration of the allosteric activator such that

$$\mathrm{V} = V_{Forward}(1 + \frac{[Act]}{K_{act}}) \quad (15)$$

For inhibitory allosteric interactions this term is present the denominator of the rate[26]. In summary we tested/trained different models using four different rate laws including different combinations of allosteric interactions including:

I. *GDP* and *GTP* allosterically activate the enzyme ***UPRT***
II. *AMP* allosterically inhibits the enzyme ***APRT***
III. *GTP* allosterically activates the enzyme ***UMPK***
IV. *UTP* allosterically inhibits the enzyme ***UMPK***

Notably for the model featured in the main text shown in equation 8 additional we demonstrate the model cannot be coarse grained further, specifically we show different that conversions from nucleobase to monophosphate, from mono to diphosphate and di to triphosphate cannot considered irreversible by removing $V_{reverse}$ from the model whereas others can.

2.1.1 Training models with alternative rate laws

To find the right model we need to balance the mechanistic detail present in the description and map the effect of the uncertainty about the parameters that are estimated, does increased parameterization lead to overfitting and a reduction in predictive power? The full model which is 'least wrong' would map individual states using general mass action kinetics. This would include the forward and reverse rate of the enzymes and their complexed intermediate states, this option is discarded out of hand as it would include too many states and rates that cannot be observed. We tested 3 different rate laws with a bireactant mechanism, BiBi ordered equilibrium reactions (equation 13), BiBi rapid equilibrium random (equation 12), a ping pong reaction (equation 14). Each assuming differences in the manner in which enzyme substrate complexes are formed and the release of the product. Regardless, all rate laws principally map onto a 'velocity curve' that

describes the rate of product formation with respect to the input (and there will be a degree of overlap here). Then, for each of the rate laws we built a model that includes allosteric interactions including either;

- Interaction I & II & III & IV
- Interaction I OR II OR III OR IV
- No interaction.

This means model descriptions including either a single allosteric interaction or none of the allosteric interactions, are nested descriptions of the model containing all interactions. When we fit the 3 largest possible models to all the data, we find the following in Fig S5.

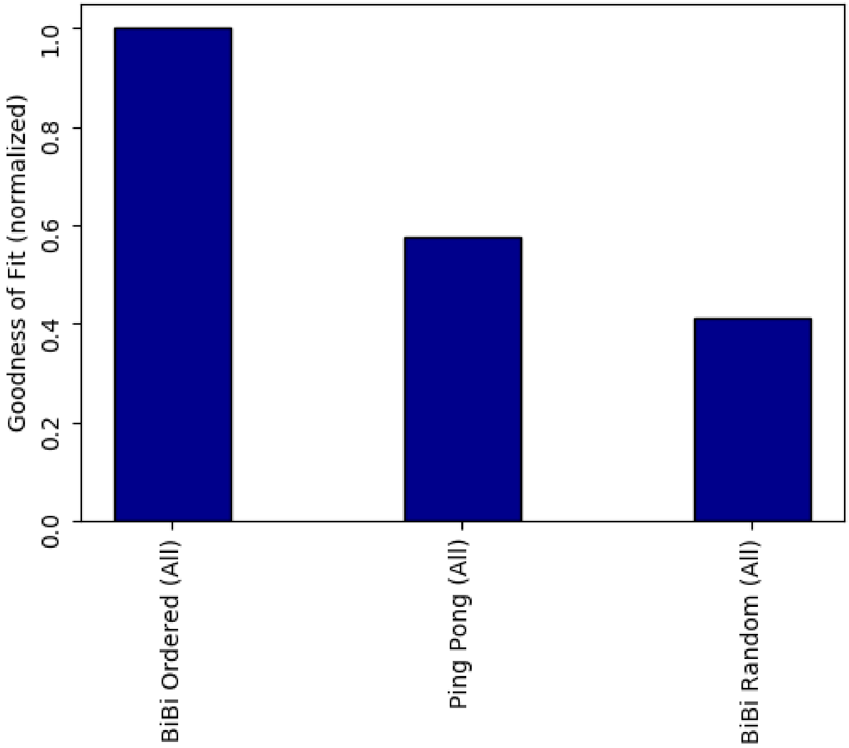

Fig. S5. This figure shows the relative goodness of fit for each of the models (see figure 3 of the main text for an explicit example of the fit). Notably the Bibi ordered equilibrium –though it has the same number of parameters- fits worse than a ping pong model The models include all of potential allosteric interactions.

The ordered reaction (eq. 13) has the worst goodness of fit score. The BiBi ping pong rate is approximately is half of that, and the BiBi rapid equilibrium random reaction slightly improves on this further. This eliminates the ordered sequential reaction rate law as suitable; it has the same number of parameters as the ping pong rate law but cannot approximate the nearly data as well. Therefore, this rate law and its nested (fewer allosteric interactions) description likely do not map onto the product formation velocities. The ping pong and BiBi rapid equilibrium random reaction fit better. The fact that the latter fits that data better is not necessarily a reflection of the model's predictive power at this point, since the BiBi reaction has more parameters with to the addition of a Ka and Kai term. This means it has more degrees of freedom to fit the data. To assess if it has over fitted the data (biased training) we have to see how it performs when it predicts test data, to demonstrate we opted to show the results in light of Figure 5 of the main text (predicting triphosphate blend).

2.1.2 Testing models with alternative rate laws

Having tested the goodness of fit of the full models (and eliminated a candidate), we continue with the models stripped of all allosteric interactions (their nested version). Figure S6 shows the average prediction accuracy for the experiments performed in Figure 5 of the main text using the best parameter estimates. Notably the BiBi ping pong model does not perform better than the BiBi rapid equilibrium random reaction. This suggests that the latter has not been overfit and the combination of estimated rates do map onto the kinetic landscape and is therefore a suitable model (the best). As expected, the BiBi ordered reaction performs the worst.

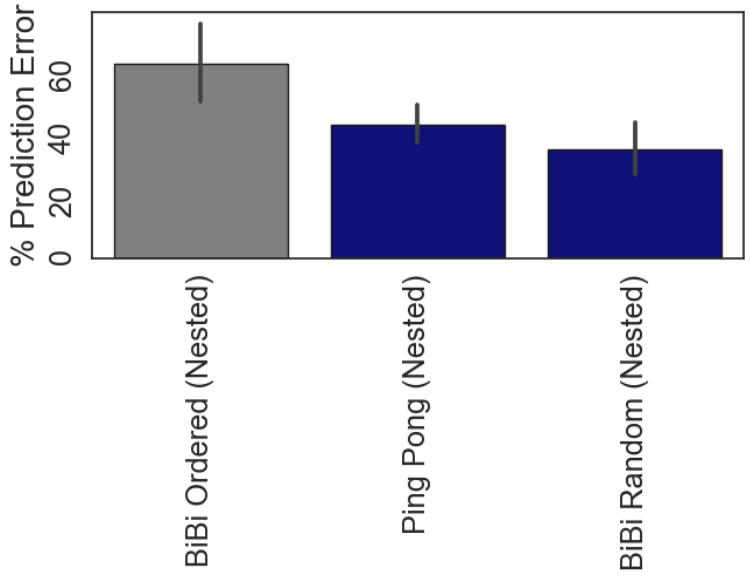

Fig. S6. Shows the average prediction error (N=20) for the conditions shown in figure 5 of the main text, notably the BiBi rate with more parameters has the lowest average prediction error, hinting that this inclusion of a Ka and Kia rate (equation 13) map onto the kinetic landscape (no overfit) in spite of the remaining uncertainty about their true value.

To assess if allosteric interactions contribute significantly to the product formation flux and thus play a significant role in shaping the time course data we include them in the best model, the BiBi rapid equilibrium random model, and compare the predictions.

2.1.3 Allosteric interactions do not significantly affect product formation fluxes

Figure S7 subsequently shows the predictive power of the BiBi random rapid equilibrium model and its potential allosteric interactions. The results indicate that the inclusion of allosteric interactions does not improve the predictive power of the model. The model that includes all potential allosteric interactions results in the worst prediction (though marginally). The additional uncertainty that stems from the inclusion interaction parameters likely leads to more overfitting and worse predictions. Models containing a singular allosteric interaction do not increase the predictive power of the model either. This means, that although allosteric interaction with enzymes coupled to beads might be present (and overfitting is less of a concern because of the optimally designed experiments), these interactions do not impact the production flux enough to compensate for the aforementioned additional uncertainty that comes with the inclusion of another parameter.

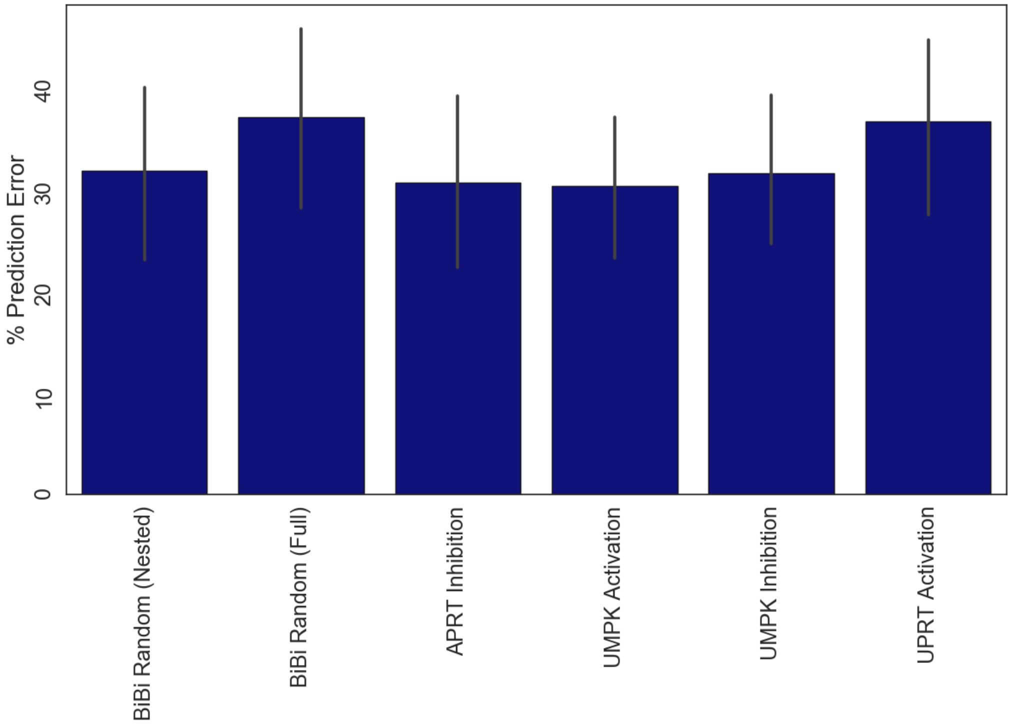

Fig. S7. Shows the prediction error of the models akin to figure S6 (N = 20), but tests the inclusion of different allosteric interaction instead of different rate laws. The predictive power of the models with allosteric interactions do not improve the predicitve power.

2.1.4 Further coarse graining the model, comparing the minimal form of the generalized rate law and BiBi Random equilibrium rate law

Finally, we explored if the BiBi random rapid equilibrium model could be reduced further. To achieve this we utilize a generalized rate equation for systems biology described in ref 30, they describe a generalized hill type rate law and effectively assumes $K_{ai}=K_a$. They demonstrate that this rate law maps onto the veloscity curves that describe the production fluxes aptly and practically differes little from the cannonical descriptions. For our system the mechanistic description the BiBi rapid equilibrium random reaction simplifies to equation (8).

Additionally, we found that for both models the reactions catalysed by the *UPRT* and *APRT* enzyme can be considered unidirectional and do not need to be included in either model for them to have predicitive power (the next section will compare reversibility).

Figure S8 compares the result of the prediction for the generalized model and the BiBi rapid equilibrium random model, including the assumed unidirectionality of the reaction of the *UPRT* and *APRT* enzyme, (this is the prediction error for the average of the experiments, the two experiments for each condition are shown in section 3.3 table S1). Figure S9 subsequently shows the difference in predictive power i.e. percentage by which the prediction error is reduced compared to the other model. Notably, although the average prediction error does not differ greatly, for the 5 conditions with higher input substrate concentrations the generalized model performs alot better, for the low substrate conditions it is only slightly worse.

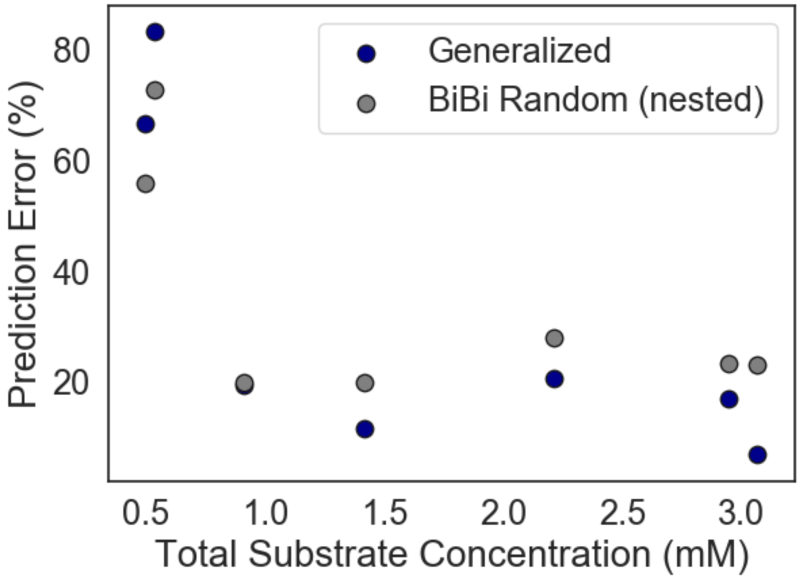

Fig. S8. Shows the average prediction of the generalized model and the BiBi rapid equilibrium random model (N= 20). As with figure 5 in the main text the x-axis shows the total substrate inflow concentration. The y-axis the prediction error expressed as the percentage the average model prediction deviates from the experimental data.

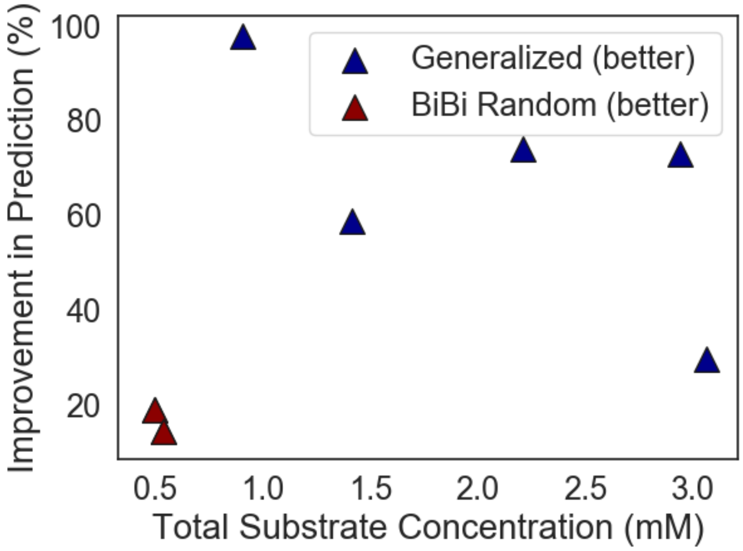

Fig. S9. Shows the difference between the prediction accuracy between the two models expressed as a percentage. Thus for the five high susbtrate conditions the generalized model is approximately 70% more accurate than the BiBi random model.

2.1.5 Demonstrating the limit of the coarse graining process.

Finally, we investigate the assumed reversibility of the reaction catalysed by the enzymes and we highlight the limits of reducing the generalized model further. In Fig. S10 we compare the predictive power of two models trained on all experimental data which not include reversibility for a specific class of reactions.

These models assume the following; the first model assumes that the conversion of only diphosphates to triphosphates (*PK*) is irreversible. The model second model assumes that the conversion of only the monophosphates to diphosphates is irreversible (*GMPK*, *UMPK*, *AK*). It is known in literature that the pyrimidine salvage pathway can operate in the opposite direction under the right conditions (and can play a role in disease pathology).[28,29] Assuming that the conditions in the reactor or the hybridization of enzymes to beads might interfere with the mechanistic function of the pathway we thought it worthwhile to explore. The fact that neither model can predict new outcomes and we observe significantly larger prediction errors especially when we assume *PK* is irreversible even though it is trained on the same data; indicates that reverse reactions are indeed, occurring. This significantly differs compared to previous model comparisons; whilst the introduction of allosteric terms, or testing different rate laws reduces the predictive power it only does so slightly in comparison. **In contrast, assuming irreversibility for these specific reactions breaks the model, it no longer has quantitative predictive power.**

When we assume *PK* converts substrates in an irreversible manner the prediction -in absolute concentrations- is off by almost an order of magnitude. When we assume that the conversion of monophosphates is irreversible it still performs worse than the original model trained on a single iteration of the OED cycle (though less bad than *PK* irreversibility).

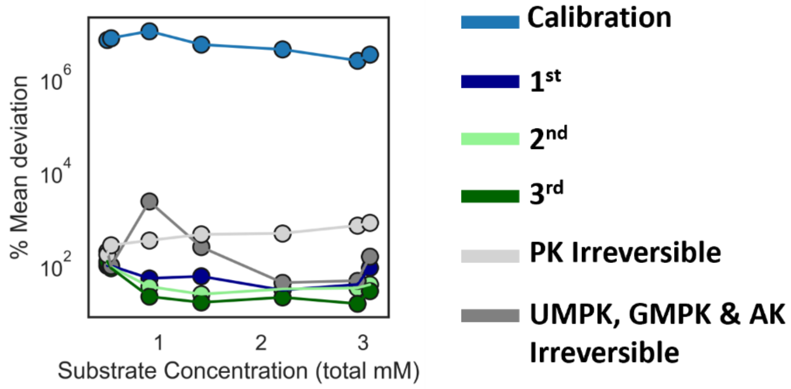

Fig. S10. The figure shows the mean deviation of the prediction from the mean for different version of the model (trained on different dataset). It includes the full model trained on just the calibration data and each subsequent iteration of the optimization cycle; this neatly shows the predictive power of the model increases as more data is added to the dataset. The figure also includes (grey lines) of 2 coarse grained models (PK or UMPK, GMPK and AK are deemed irreversible) trained on the full dataset. Assuming irreversibility breaks the model and results in significantly worse predictions.

Finally, the addition of OED cycles to the dataset is demonstrated explicitly in Fig S11, it highlights the reduction in the uncertainty about the prediction after each iteration for the model shown in equation 8.

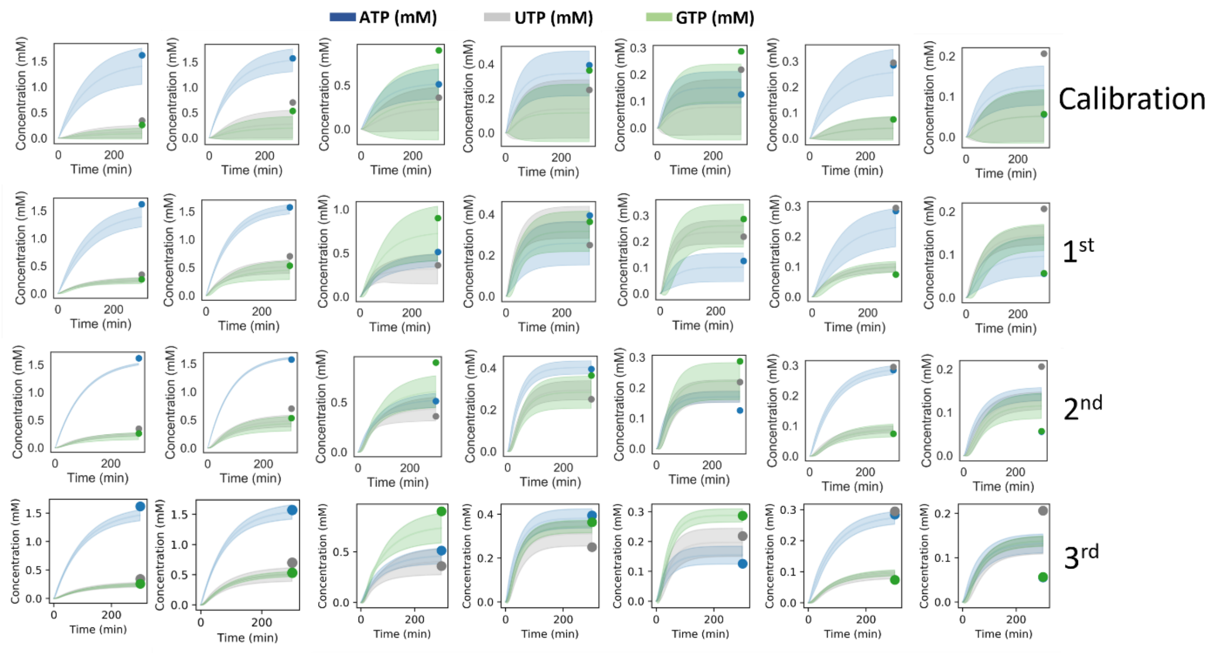

Fig. S11. Shows the prediction of the final blends for each version of the dataset/iteration of the cycle from the calibration to the final experiment. As more data is added each iteration (number on the right) we reduce uncertainty around the predicted yield (shaded area). The y-axis shows the concentration in mM, the x-axis the time in minutes. The concentrations of ATP (blue), UTP (grey) and GTP (green) are shown.

2.2 A note on parameter estimation methods

To rapidly estimate the parameter likelihoods and check if our algorithm converges to appropriate solutions we tested another method, pyMC. It uses the sensitivities to fit rates with a gradient descent-based method. We previously demonstrated that this works well with steady state data. For this dataset however we observed poor convergence and the parameter estimates were not useful. We hypothesise that the perturbations make the fitness landscape very non-convex, including a lot of local minima that do not map onto the production fluxes of the thriphosphates. In Fig S12 below shows the fit using the pyMC gradient descent-based methods to obtain parameter likelihoods, we utilized the fits of the previous algorithm as starting points to for the estimation. This improved the convergence but makes the method less useful, though the model did converge to most observed species, some species such as UMP diverge.

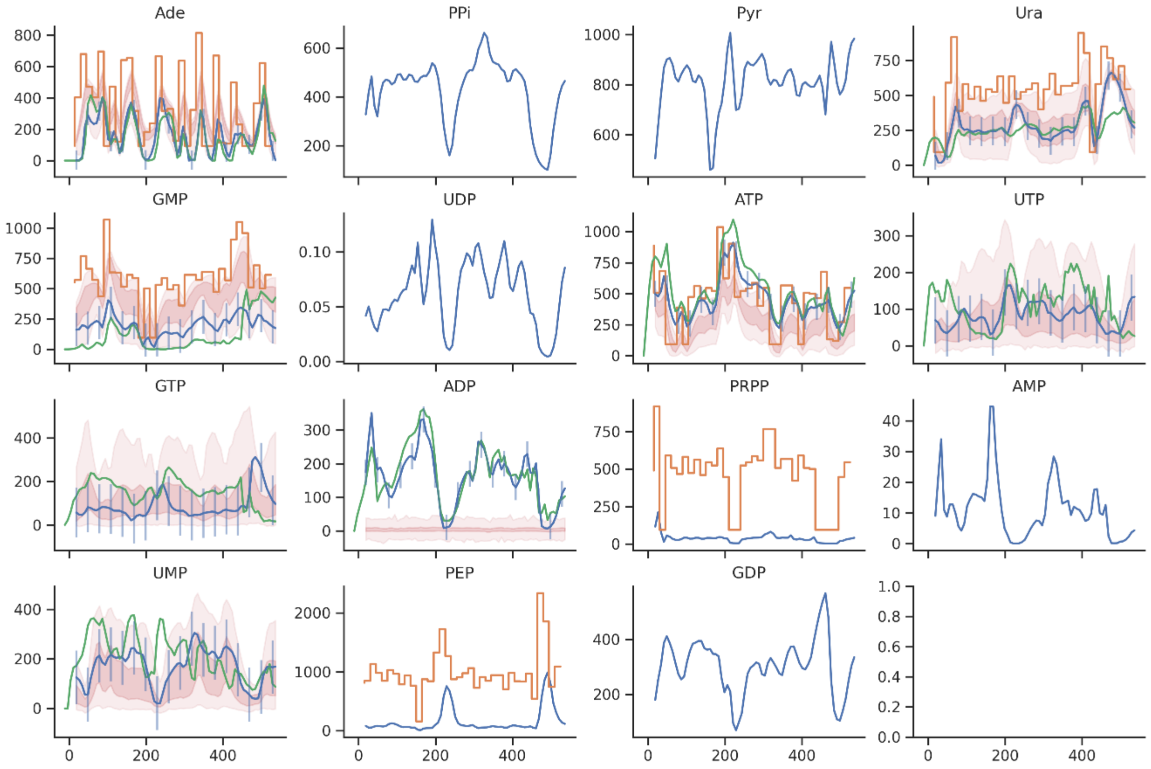

Fig S12. Fit using pyMC after using the estimates from as input shows improved compared to ab initio optimizations. The blue line is the simulation, the green line the data, the orange line is the input.

2.3 Effect of Different Flow Rates on Product Formation

In the final experiment of the OED cycle shown in the main text we vary the flow rate. By varying the total flow rate, we can control conversion, favouring monophosphates (fast flow rates), diphosphates (medium flow rates), triphosphates (low flow rates). We did not alter these flowrates drastically within short time frames as this could introduce additional extrinsic noise both within the reactor and when the samples are collected from the CSTR. Thus, we opted to introduce three total flowrates wherein we vary the substrate concentrations. The effect on the conversion of the input can be seen in Fig S13 and S14. It shows the mean and distribution of the monophosphates and input bases, the diphosphates and triphosphates.

This is a rough representation, but it shows that a transition from monophosphates to triphosphates can be observed as the flow rate is decreased, this increases the information within the experiment as Baltussen *et al.*[2] have shown. Intuitively, knowing that there is a total flow rate wherein a substrate is not converted

means there is a limit to the speed an enzyme can convert it e.g., between 150-300 ul/h all the nucleobases are converted, this means we do not know how fast enzymes have converted the substrates, only that occurred faster than dilution. Conversely at 600 ul/h, nucleobases are still present, this means conversion is slower than dilution. Notably it had effect, Fig S14B shows the relative mean width of all parameter distributions shrank significantly between the 2nd and 3rd final experiment. We estimate a range of rates when we train the models, we do not calculate an exact parameter likelihood, this means all parameters within this distribution, are considered to be equally likely from a goodness of fit perspective. The resulting shrinkage in the width of the distributions are therefore rough indicators of a reduction in the possible combinations of kinetic rates that can fit the data, not an exact quantification.

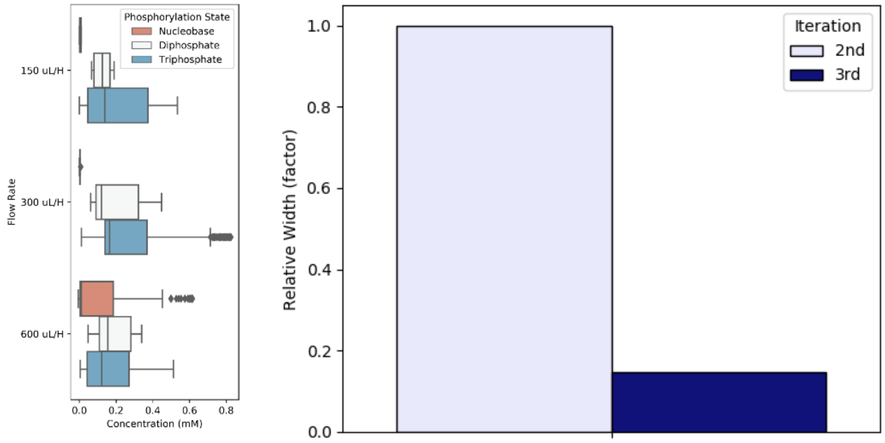

Fig. S13 & S14. (Left S13) shows the phosphorylation level of the nucleobases for different flow rates. The data is sampled by taking all datapoints across a 100-minute time window for each flow rate from the final iteration (shown in Fig. S20). (Right S14) shows the difference between the mean width of all parameters estimated between the 2nd and 3rd iteration (including different flow rates within a single experiment). The width is approximated by dividing the average of 3 largest parameters values by the 3 smallest estimated parameter values for each parameter.

3. Experimental Data

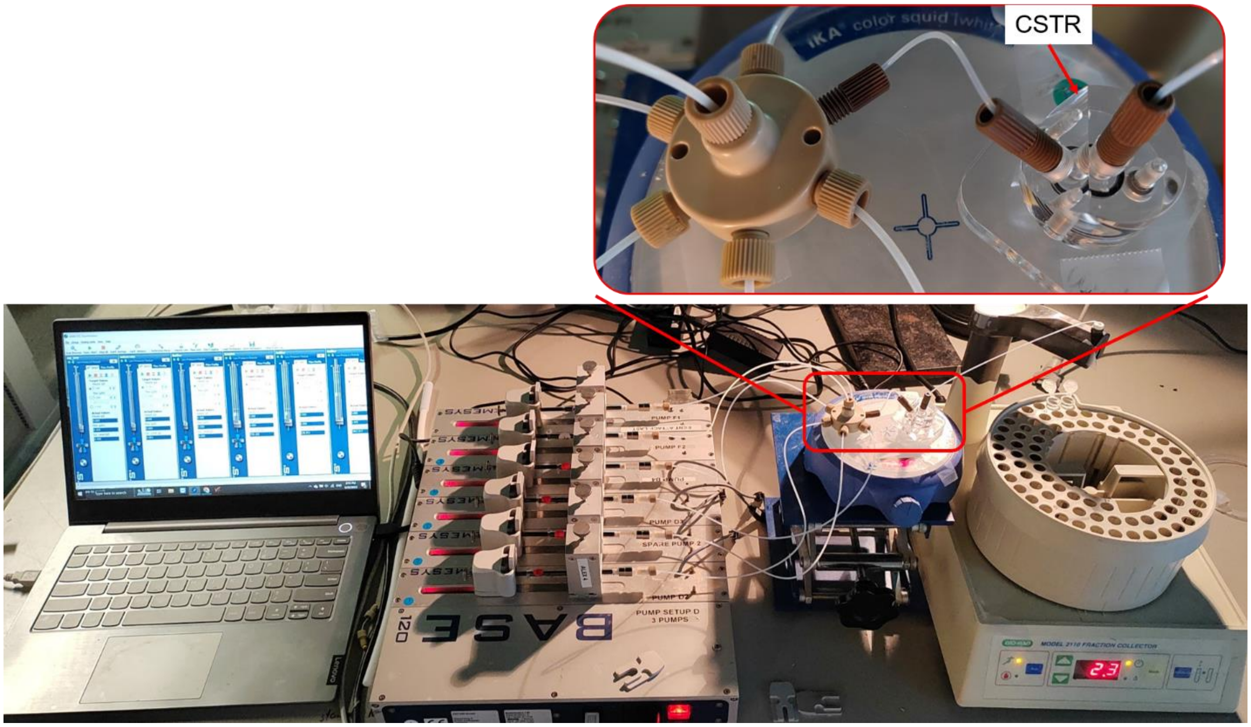

Fig. S15. Overview of the experimental flow setup.

3.1 Ion Pair HPLC Data

A total of three iterations of the optimization cycle were performed, the enzymes are fixed to the microfluidic device, each experiment has a unique enzyme concentration, we quantify their relative units (i.e. how many beads are used). First, we needed to establish the compounds were detected by ion-pair reversed-phase HPLC. Fig. S16 shows 8 peaks were separated.

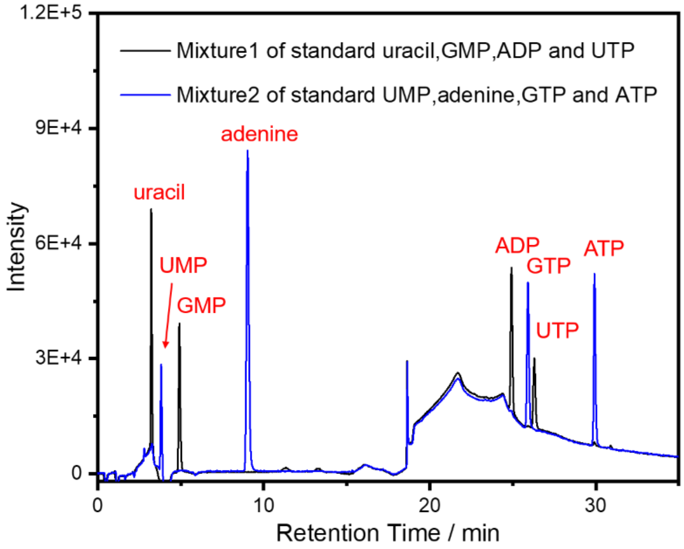

Fig. S16. Separation of eight standard nucleotides by ion-pair reversed-phase HPLC. Mixture1: 100 uM each of uracil, GMP, ADP and UTP; Mixture2: 100 uM each of UMP, adenine, GTP and ATP. Injection volume: 1 ul. Detection: 254 nm. See the detailed elution method in method part.

3.2 Flow Experiment Data

The first experiment was subsequently designed manually (calibration) and is largely utilized to probe the boundaries of the system specifically: for which range of input concentrations can conversion be observed and can differentiate between causes when nothing is observed i.e., determining whether this due to unfavourable kinetics or because the limits of detection reached (we know the enzymes work as for each we performed an activity assay in Section 3.3). The results are shown in Fig. S17. In the first iteration of the optimization cycle, we maintain a constant total flow, changing the individual flow rates of the input substrates within these bounds (Fig. S18), the latter 2 iterations in the cycle make use of a dynamic flow profile where the total flow transitions between 4 states (Fig. S19 and Fig. S20).

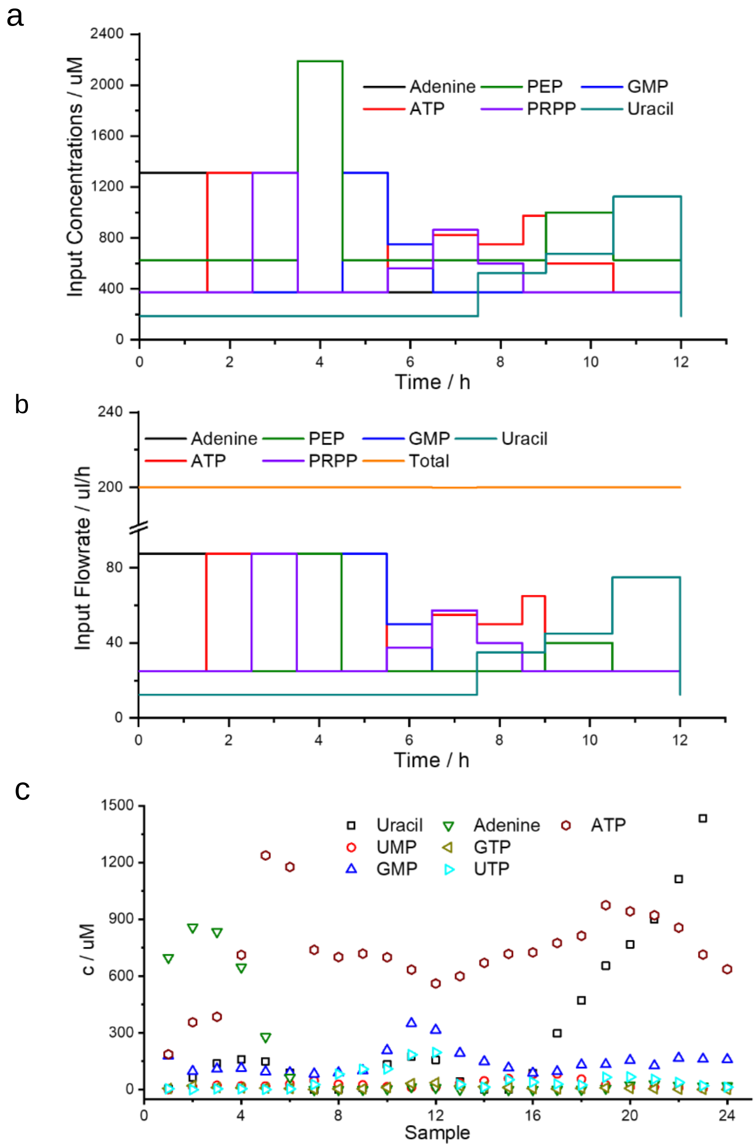

Fig. S17. a) shows input concentrations into the reactor over time for the calibration experiment, b) shows the inflow rate of each syringe over time (and the total flow rate), c) shows the concentrations as measured with the ion-pair HPLC in uM. The relative enzyme concentrations defined as ul of beads are AK: 2 ul, PK: 20 ul, UMPK: 15 ul, APRT: 2 ul, GMPK: 5 ul, UPRT: 15 ul.

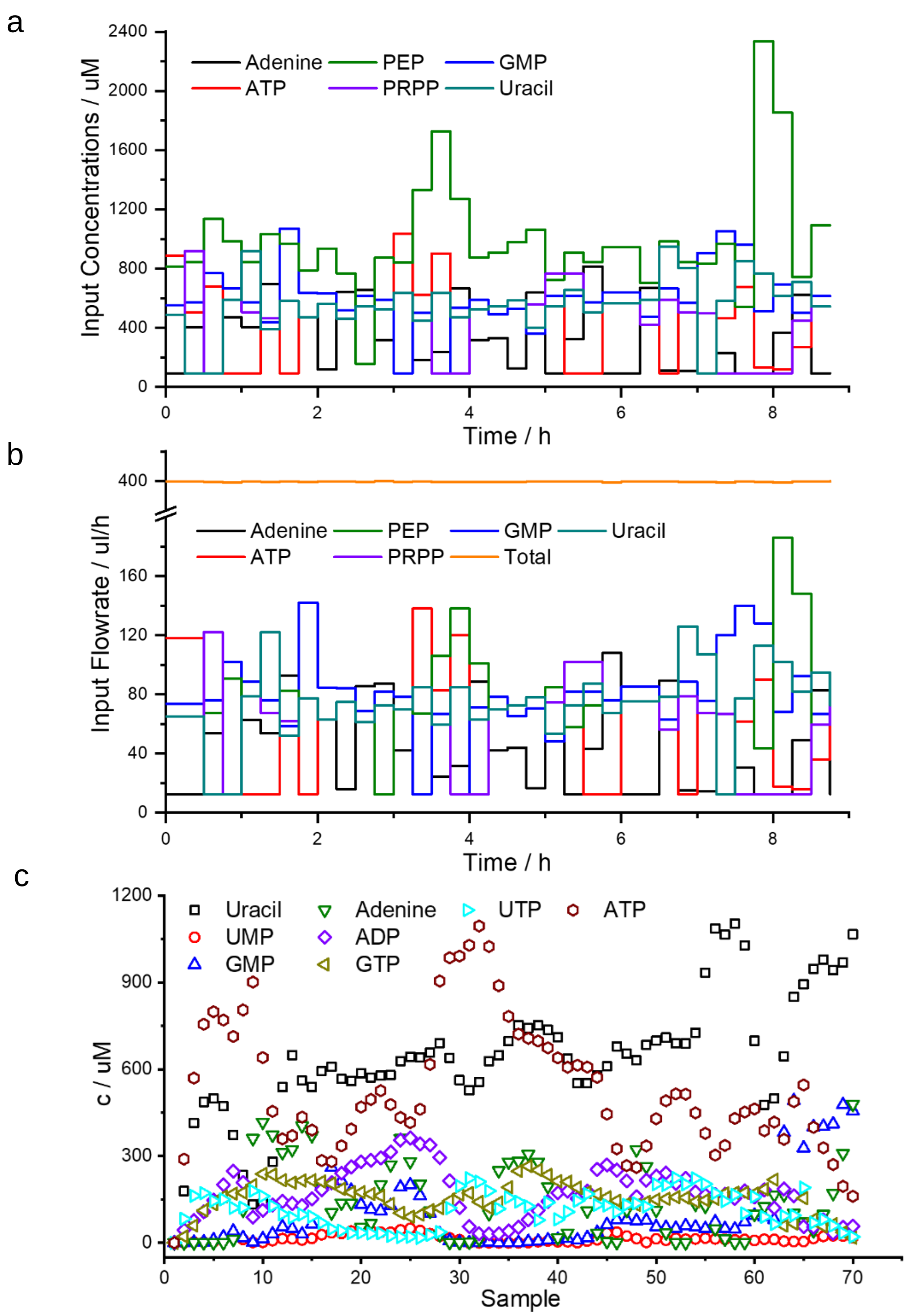

Fig. S18. a) shows input concentrations into the reactor over time for the first flow experiment, with constant flow, b) shows the inflow rate of each syringe over time (and the total flow rate), c) shows the concentrations as measured with the ion-pair HPLC in uM. The relative enzyme concentrations are AK: 2 ul, PK: 20 ul, UMPK: 15 ul, APRT: 2 ul, GMPK: 5 ul, UPRT: 15 ul.

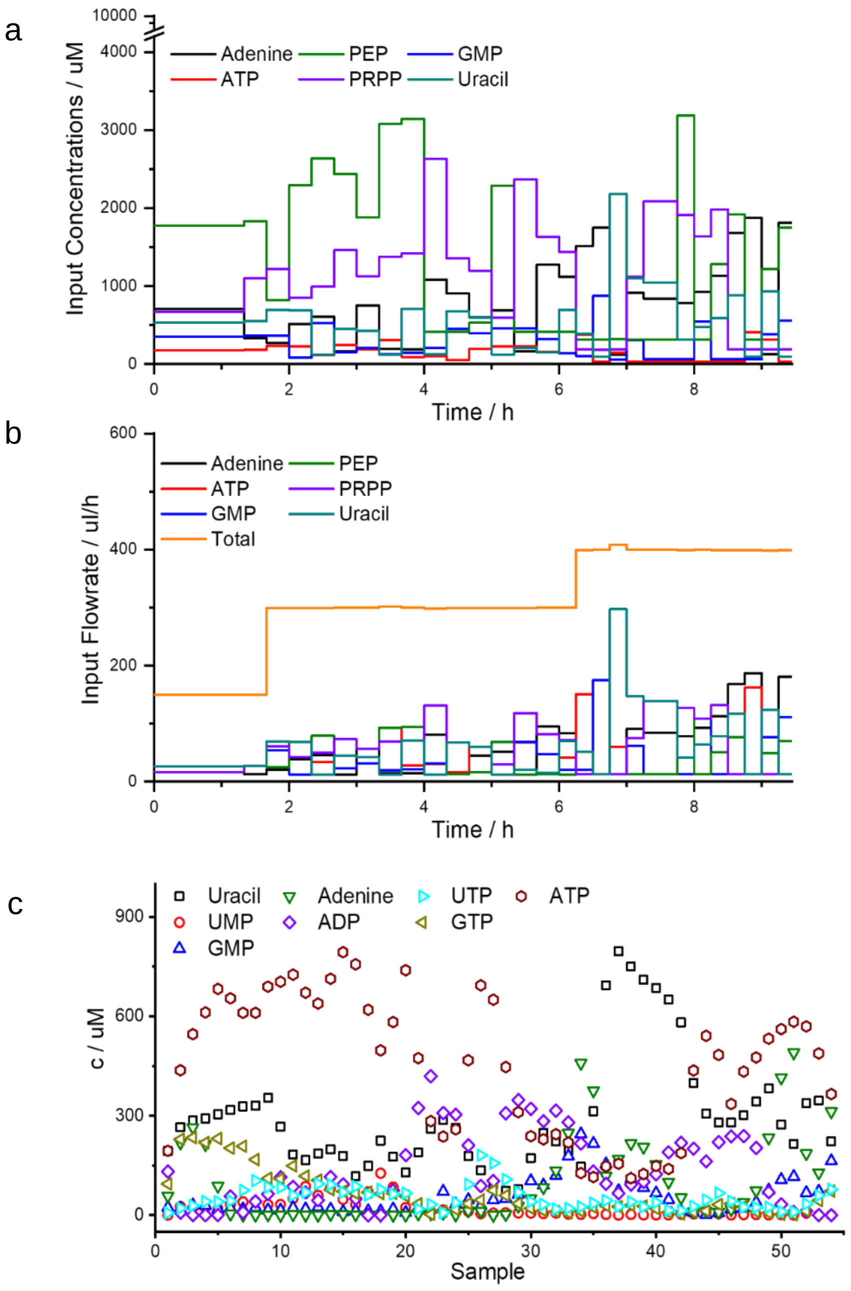

Fig. S19. a) shows input concentrations into the reactor over time for the second flow experiment with a dynamic 3 tier flow profile. b) shows the inflow rate of each syringe over time (and the total flow rate), c) shows the concentrations as measured with the ion-pair HPLC in uM. The relative enzyme concentrations are AK: 4 ul, PK: 38 ul, UMPK: 8 ul, APRT: 5ul, GMPK: 3 ul, UPRT: 13 ul.

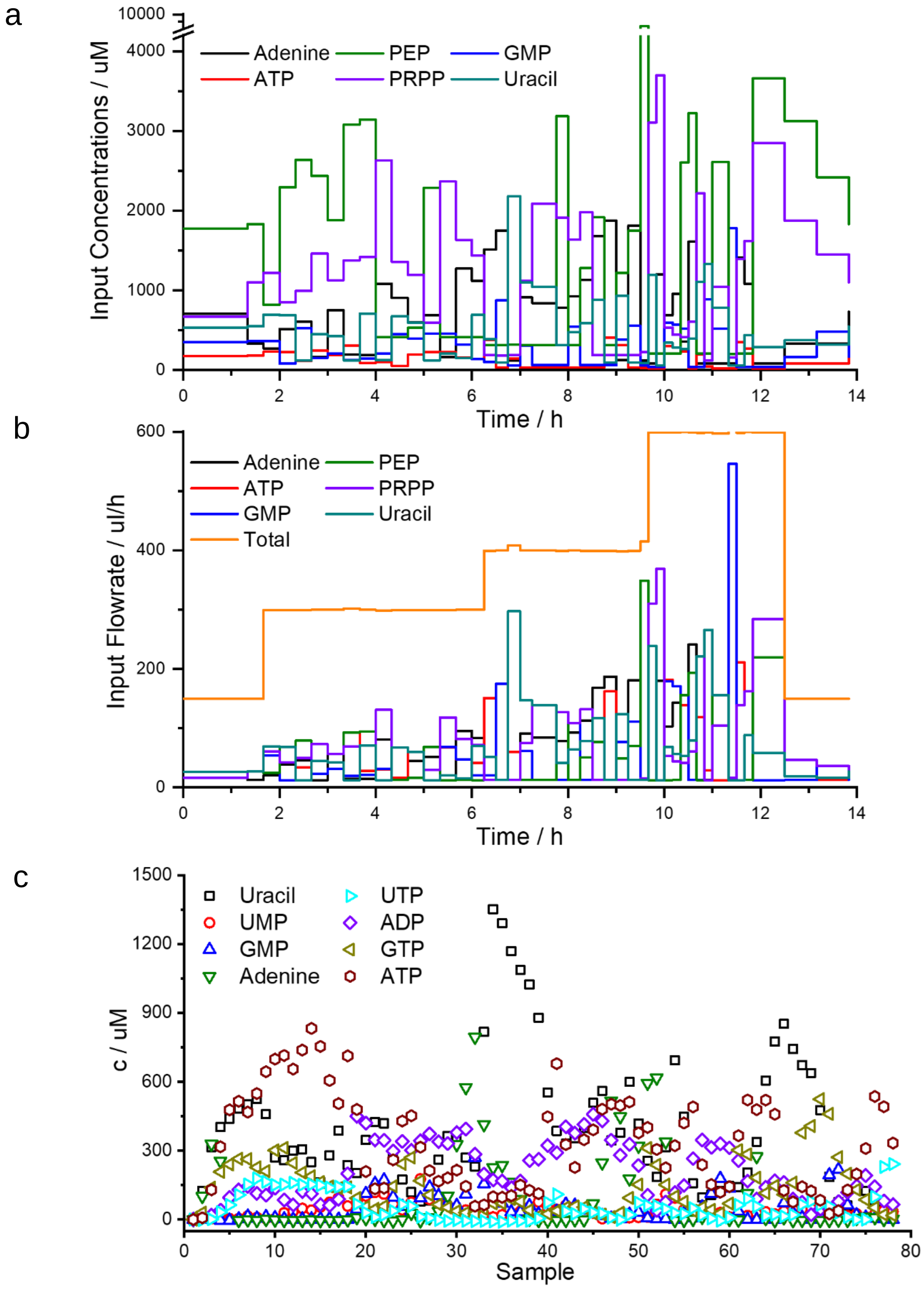

Fig. S20. a) shows input concentrations into the reactor over time for the first flow experiment, with a dynamic 4 tier flow profile. b) shows the inflow rate of each syringe over time (and the total flow rate), c) shows the concentrations as measured with the ion-pair HPLC in uM. The relative enzyme concentrations are AK: 1.5 ul, PK: 20 ul, UMPK: 40 ul, APRT: 2.5 ul, GMPK: 10 ul, UPRT: 6 ul.

3.3 Output control experiments

Table S1. The kinetic model designed experimental conditions to produce the on-demand outputs. Volume of Enzyme-beads: APRT 4ul; AK, 4ul; PK, 30ul; GMPK, 3.75ul; UPRT, 12 ul; UMPK, 33.75ul.

	**Input** (uM)						
**Exp**	***Uracil***	***Adenine***	***GMP***	***PRPP***	***PEP***	***ATP***	**Flowrate** (ul/h)
1	641	225	647	3000	2964	1431	72
2	296	564	311	2747	2354	1040	60
3	1231	207	1198	698	3000	430	72
4	496	290	484	671	2169	147	144
5	298	130	424	435	1476	560	162
6	164	100	182	765	940	52	96
7	109	167	124	1472	1116	134	72

Table S2. Results of the final experiments

	**Output_1** (uM)			**Output_2** (uM)			**Output_Average** (uM)					
**Exp**	***UTP***	***GTP***	***ATP***	***UTP***	***GTP***	***ATP***	***UTP***	**std**	***GTP***	**std**	***ATP***	**std**
**1**	700	531	1573	769	580	1580	735	35	556	25	1577	4
**2**	342	257	1617	353	287	1502	348	6	272	15	1560	58
**3**	360	900	513	380	1063	489	370	10	982	82	501	12
**4**	250	364	395	337	332	422	294	44	348	16	409	14
**5**	219	287	125	265	212	85	242	23	250	38	105	20
**6**	206	57	55	197	148	149	202	5	103	46	102	47
**7**	295	74	284	116	53	149	206	90	64	11	217	68

4. Enzyme Purification and Validation of Enzyme Activity

4.1 Enzymes: Source and Availability

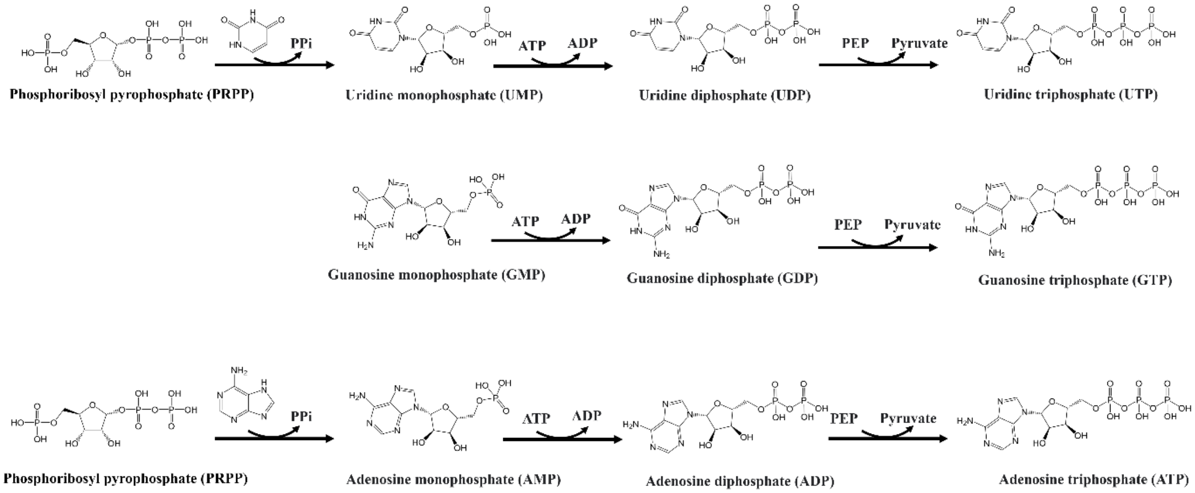

Fig. S21. Detailed overview of the nucleotide salvage module with the molecular structures of the substrates and reaction products; PEP, phosphoenolpyruvic acid.

Some enzymes (figure S21) were cloned into and purified from *E. coli*; others were purchased from Sigma (Table S3). This part of the SI contains protocols for the purification process and the characterization of enzyme activity after coupling them to beads to assess if they still function. Note that we quantify the activity of the beads and can account for their concentration, not individual enzymes. Thus, in the model we use relative concentrations, choosing to set the calibration experiment as a reference.

Table S3. Overview of the enzymes used in the pyrimidine salvage pathway

Enzyme	Source	EC	Origin	Abbreviation	Reaction
Adenine phosphoribosyl transferase	ref 34	2.4.2.7	*E. coli*	*APRT*	*PRPP + adenine = AMP + PPi*
Adenylate kinase (Myokinase)	Sigma (M3003)	2.7.4.3	Rabbit muscle	*AK*	*AMP + ATP = ADP + ADP*
Guanylate kinase	ref 35	2.7.4.8	*E. coli*	*GMPK*	*GMP + ATP = ADP + GDP*
Uridylate kinase	ref 36	2.7.4.22	*E. coli*	*UMPK*	*UMP + ATP = ADP + UDP*
Uridine phosphoribosyl transferase	ref 34	2.4.2.9	*E. coli*	*UPRT*	*PRPP + uracil = UMP + PPi*
Pyruvate kinase	Sigma (P1506)	2.7.1.40	Rabbit muscle	*PK*	*ADP + PEP = ATP + Pyruvate*<br>*GDP + PEP = GTP + Pyruvate*<br>*UDP + PEP = UTP + Pyruvate*

4.2 Cloning, expression and purification

4.2.1 UMPK

The gene for *UMPK* was amplified from Escherichia coli K12 derived XL-1 blue competent cells using primers:

UMPK-Fw 5’GCATGACGTAGTACATATGGCTACCAATGCAAAACCCGTCTATAAACGC3’
UMPK-Rv 5’ACTGGTACGCCTCGAGTTATTCCGTGATTAAAGTCCCTTCTTTTTCACCC3’.

Sequences in bold and underlined indicate the restriction sites for subsequent cloning into a pET15b expression vector. The PCR mixture contained 1 µM of each primer, 0.2 mM dNTPs, 0.25 ul of competent *E. coli* cells and 2 U of Pfu DNA polymerase in its standard reaction buffer. Mixture was cycled for 25 times between 95°C (30s), 58°C (30s) and 72°C (2min). Agarose gel analysis of the reaction product showed a single band around 725 bp as expected. DNA was digested with NdeI and XhoI, purified over agarose gel using a Qiaquick gel extraction kit (Qiagen) and subsequently ligated into a dephosphorylated NdeI + XhoI digested pET15b vector introducing an N-terminal hexahistidine tag. Recombinant plasmids were checked on insert and subsequently transformed into *E. coli* BL21(DE3) for protein expression. Bacteria were cultivated in 1 liter of LB media at 30°C to OD600nm ~0.6 upon which protein expression was induced by adding IPTG to 0.5 mM. Cells were further cultivated overnight at 18°C and then harvested by centrifugation for 10 minutes at 5000 rpm in a Beckman JA-10 rotor. All subsequent steps were at 4°C unless otherwise noted. The pellet was resuspended in 40 mL buffer A (50 mM Na2B4O7.10H2O, 500 mM NaCl, 0.5 mM Na2-EDTA, 5 mM β-mercaptoethanol at pH 8.5) containing 20 mM imidazole, 20 µg/mL PMSF and 1 mg/mL lysozyme. The cell suspension was sonicated for 30 seconds at 17 micron amplitude for 6 times with pause intervals of 1 minute on ice. Lysate was centrifuged for 30 minutes at 17000 rpm in a Beckman JA25.50 rotor. Cleared supernatant was loaded onto a 5 mL Ni2+-sepharose HP column (GE Healthcare) that was pre-equilibrated in buffer A containing 20 mM imidazole. The column was washed with 25 ml of buffer A containing 50 mM imidazole and bound proteins were eluted with buffer A containing 500 mM imidazole and collected in fractions of ~1 mL. Fractions were analyzed on SDS-PAGE and fractions containing solely *UMPK* (8 in total) were pooled and dialyzed in a 3.5 kDa MWCO dialysis tubing against 1 liter of 20 mM potassium phosphate buffer, pH 7.2, refreshing the dialysis buffer once. Enzyme isolate became opaque and was centrifuged for 1 minute at 14000 rpm in a table top centrifuge to remove any formed precipitate. Opaque supernatant was pipetted into a fresh tube and stored at 4°C.

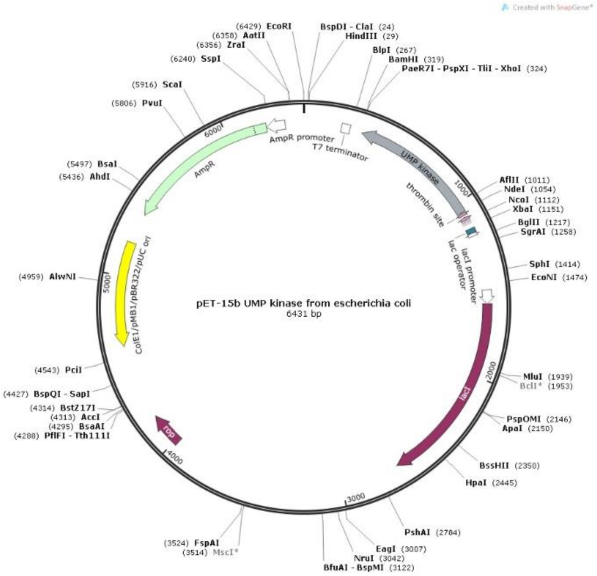

Fig. S22 Vector Map of UMPK plasmid.

4.2.2 GMPK

The gene for *GMPK* was amplified from *Escherichia coli* K12 derived XL-1 blue competent cells using primers:

GMPK-Fw 5’GCATGACGTAGTA**<u>CATATG</u>**GCTCAAGGCACGCTTTATATTGTTTCTGCCC3’
GMPK-Rv 5’ACTGGTACGC**<u>CTCGAG</u>**TCAGTCTGCCAACAATTTGCTGATTAAAGCGTC3’.

Sequences in bold and underlined indicate the restriction sites for subsequent cloning into a pET15b expression vector. The PCR mixture contained 1 µM of each primer, 0.2 mM dNTPs, 0.25 ul of competent *E. coli* cells and 2 U of *Pfu* DNA polymerase in its standard reaction buffer. Mixture was cycled for 25 times between 95°C (30s), 60°C (30s) and 72°C (2min). Agarose gel analysis of the reaction product showed a single band around 725 bp as expected. DNA was digested with *Nde*I and *Xho*I, purified over agarose gel using a Qiaquick gel extraction kit (Qiagen) and subsequently ligated into a dephosphorylated *Nde*I + *Xho*I digested pET15b vector introducing an N-terminal hexahistidine tag. Recombinant plasmids were checked on insert and subsequently transformed into *E. coli* BL21(DE3) for protein expression. Bacteria were cultivated in 0.5 liter of LB media at 30°C to OD_{600nm} ~0.6 upon which protein expression was induced by adding IPTG to 0.5 mM. Cells were further cultivated overnight and then harvested by centrifugation for 10 minutes at 5000 rpm in a Beckman JA-10 rotor. All subsequent steps were at 4°C unless otherwise noted. The pellet was resuspended in 40 mL buffer A (100 mM potassium phosphate buffer at pH7.5, 300 mM NaCl, 1 mM Na_2-EDTA and 5 mM β-mercaptoethanol) containing 20 mM imidazole, 20 µg/mL PMSF and 1 mg/mL lysozyme. The cell suspension was sonicated for 30 seconds at 17 micron amplitude for 5 times with pause intervals of 1 minute on ice. Lysate was centrifuged for 45 minutes at 16000 rpm in a Beckman JA25.50 rotor. Cleared supernatant was loaded onto a 5 mL Ni^{2+}-sepharose HP column (GE Healthcare) that was pre-equilibrated in buffer A containing 20 mM imidazole. The column was washed with 25 ml of buffer A containing 50 mM imidazole and bound proteins were eluted with buffer A containing 300 mM imidazole and collected in fractions of ~1.5 mL. Fractions were analyzed on SDS-PAGE and fractions containing solely *GMPK* (5 in total) were pooled and dialyzed in a 3.5 kDa MWCO dialysis tubing against 1 liter of 20 mM potassium phosphate buffer, pH 7.2, refreshing the dialysis buffer once. Enzyme isolate was centrifuged for 1 minute at 14000 rpm in a table top centrifuge to remove any formed precipitate. Supernatant was pipetted into a fresh tube and stored at 4°C.

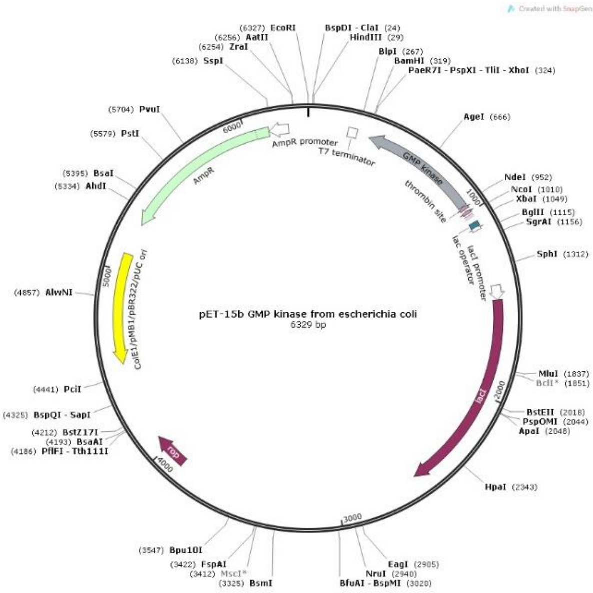

Fig. S23 Vector Map of GMPK plasmid.

4.3 Enzyme immobilization

4.3.1 Empty hydrogel beads preparation

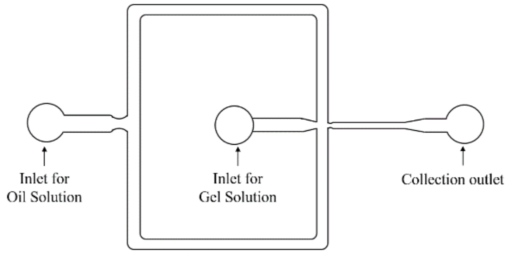

Figure S24 Schematic of the microfluidic device for hydrogel beads production.

The microfluidics device (Fig.S24) used to produce empty hydrogel beads was fabricated as reported[31]. The gel solution contained 9.6% (w/v) acrylamide, 0.4% (w/v) N,N'-methylenebisacrylamide, 0.5% (w/v) acrylic acid and 1.5% (w/v) 2,2'Azobis(2-methylpropionamidine) dihydrochloride. The oil solution was 1.5% (v/v) Pico-Surf™ 1 in fluorinated fluid HFE-7500 (3M). The flow rates for gel solution and oil solution were 600 µL/h and 900 µL/h, respectively. The outflow was collected in the Eppendorf tube where there was 100 µL mineral oil to prevent the evaporation of water. After polymerization by UV for 10 minutes at 70% gain, the empty beads were washed 3 times with 20% (v/v) 1H,1H,2H,2H-Perfluoro-1-octanol in HFE-7500 (3M), then 3 times with 1% (v/v) Span 80 in hexane, 3 times with 0.1% (v/v) Triton X-100 in water and finally 3 times with water. Then the empty hydrogel beads were freeze dried and stored in -20 ^{o}C.

4.3.2 Immobilization procedure of enzymes on empty hydrogel beads individually

10 mg Empty hydrogel beads were dissolved in 310 ul water. Then 300 ul 1-(3-Dimethylaminopropyl)-3-ethylcarbodiimide hydrochloride (500 mM), 300ul N-Hydroxysuccinimide (500 mM) and 900 ul water were added. After mixing for 30 min on a roller bank at room temperature, the mixtures were centrifuged and the supernatant was removed. Then the active beads were washed 3 times by adding water, mixing, centrifuging and removing the supernatant. Then 450 ul free enzyme solutions (*APRT, 0.45mg/ml; AK, 1.78 mg/ml; PK, 1.22 mg/ml; UPRT, 1.55 mg/ml; UMPK, 4.52 mg/ml; GMPK, 1.21 mg/ml*) was added to the beads. The mixture was put on the roller bank for 2 hours. Sequentially, enzyme-beads conjunctions were washed 8 times by adding water, mixing, centrifuging and removing the supernatant. Finally, enzyme-beads were frozen dried and stored in -20 ^{o}C.

4.4 Characterization of Enzyme-beads

4.4.1 PK beads characterization

1 ml of assay mixture contains:

- IVTT Buffer
- 0.2 mM NADH (prepare stock freshly)
- 1.5 mM PEP
- 1.5 mM ADP
- 2 U lactate dehydrogenase
- PK_beads: 6 ul

Calculate the activity of the PK_beads solution according to:

$$U = \frac{(OD_{t2} - OD_{t1}) * 1000 * V_{reaction}}{(t2 - t1) * 6220 * V_{enzyme}}$$

Where t is elapsed time in minutes, OD is the linear absorbance change over t2 - t1, V_{enzyme} is the volume of enzyme solution added in µl and $V_{reaction}$is the reaction volume in ml, 6220 is the molar extinction coefficient of *NADH* in $mol.l^{-1}cm^{-1}$ at 340 nm and U is the number of units denoted as µmol *ADP* converted to *ATP* per minute per µl of enzyme solution ($\mu mol.min^{-1}\mu l^{-1}$). Figure S25 and S26 show the activity calculation and characterization respectively.

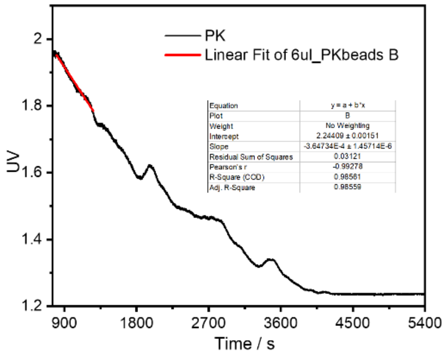

Fig. S25. progress curve of PK beads assay; The unit is calculated as 0.018 µmol per min per mg enzyme beads.

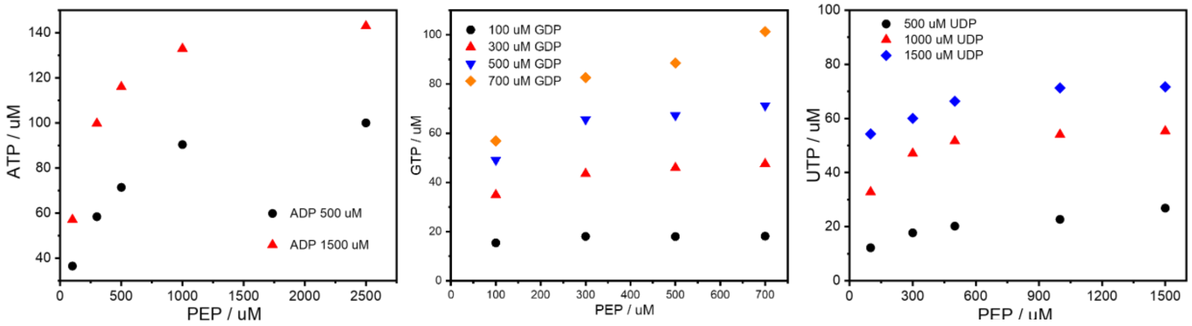

PK_beads: 2 ul **Flowrate:** 750 ul/h

ADP: 500, 1500 uM

PEP: 100, 300, 500, 1000, 2500 uM

PK_beads: 6 ul **Flowrate:** 750 ul/h

GDP: 100, 300, 500, 700 uM

PEP: 100, 300, 500, 700 uM

PK_beads: 6 ul **Flowrate:** 750 ul/h

UDP: 500, 1000, 1500 uM

PEP: 100, 300, 500, 1000, 1500 uM

Fig. S26. Activity Characterization of PK beads in flow at steady state. Enzyme PK remain active after immobilization.

4.4.2 AK beads characterization

1 ml of assay mixture contains:

- IVTT Buffer
- 0.2 mM NADH (prepare stock freshly)
- 1.5 mM Phosphoenolpyruvate
- 1.5 mM AMP
- 3 mM ATP
- 2 U pyruvate kinase
- 2 U lactate dehydrogenase
- 6 ul AK_Beads

Calculate the activity of the *AK* beads solution according to:

$$U = \frac{(OD_{t2} - OD_{t1}) * 1000 * 0.5 * V_{reaction}}{(t2 - t1) * 6220 * V_{enzyme}}$$

Like to *PK*, t is elapsed time in minutes, OD is the linear absorbance change over t2 - t1, V_{enzyme} is the volume of enzyme solution added in µl and $V_{reaction}$ is the reaction volume in ml. Figure S27 and S28 show the activity calculation and characterization respectively.

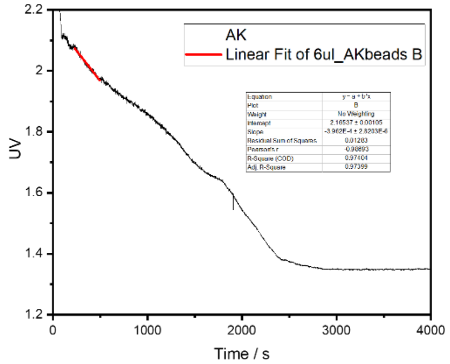

Fig. S27. progress curve of AK beads assay; The unit is calculated as 0.01 µmol per min per mg enzyme beads.

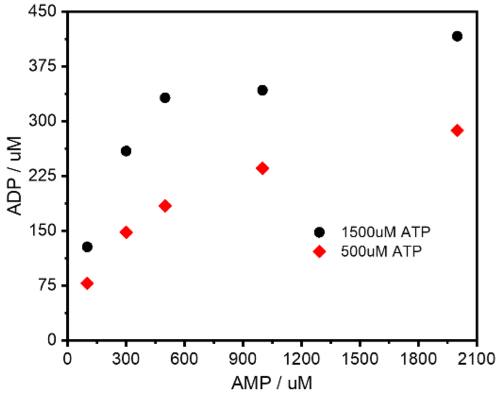

AK_beads: 4 ul **Flowrate**: 750 ul/h

ATP: 500, 1500 uM

PEP: 100, 300, 500, 1000, 2000 uM

Fig. S28. Activity characterization of AK beads in flow at steady state. Enzyme AK remain active after immobilization.

4.4.3 APRT beads characterization

1 ml of assay mixture contains:

- IVTT Buffer
- 0,2 mM NADH (prepare stock freshly)
- 1 mM Phosphoenolpyruvate
- 1,5 mM PRPP
- 1,5 mM Adenine hydrochloride
- 3 mM ATP
- 2 U lactate dehydrogenase
- 2 U pyruvate kinase
- 2 U adenylate kinase
- 8 ul APRT_beads

Calculate the activity of the APRT beads solution according to:

$$U = \frac{(OD_{t2} - OD_{t1}) * 1000 * 0.5 * V_{reaction}}{(t2 - t1) * 6220 * V_{enzyme}}$$

Like *PK*, t is elapsed time in minutes, OD is the linear absorbance change over t2 - t1, V_{enzyme} is the volume of enzyme solution added in µl and $V_{reaction}$ is the reaction volume in ml. Figure S29 and S30 show the activity calculation and characterization respectively.

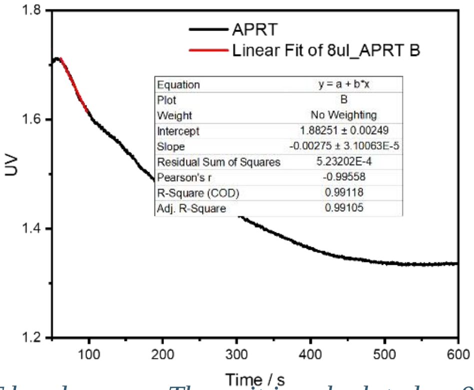

Fig. 29. progress curve of APRT beads assay; The unit is calculated as 0.051 µmol per min per mg enzyme beads.

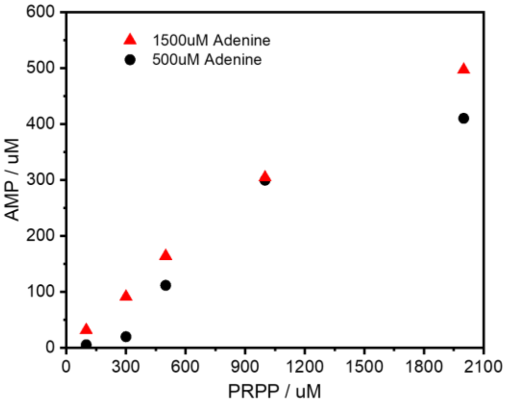

APRT_beads: 8 ul **Flowrate**: 750 ul/h

Adenine: 500, 1500 uM

PRPP: 100, 300, 500, 1000, 2000 uM

Fig. S30. Activity characterization of APRT beads in flow at steady state. Enzyme APRT remains active after immobilization.

4.4.4 UPRT beads characterization

IVTT buffer
1.5 mM PRPP
0.1 mM uracil
6 ul UPRT beads

Calculate the activity of the *UPRT* beads according to:

$$U = \frac{(OD_{t2} - OD_{t1}) * 1000 * V_{reaction}}{(t2 - t1) * 2763 * V_{enzyme}}$$

Where t = elapsed time in minutes, OD is the linear absorbance change over $t_2 - t_1$, V_{enzyme} is the volume of enzyme solution added in µl and $V_{reaction}$ is the reaction volume in ml, ε is the molar extinction coefficient in $mol^{-1}.l.cm^{-1}$ 271 nm and U is the number of units denoted as µmol uracil coupled to *PRPP* per minute per µl of enzyme solution ($\mu mol.min^{-1}\mu l^{-1}$). The absorbance is 2763 instead of 6220. Figure S31 and S32 show the activity calculation and characterization respectively.

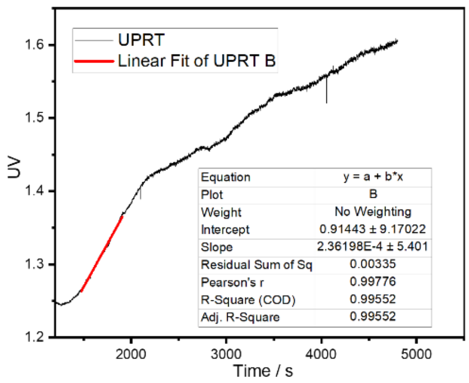

Fig. S31. progress curve of UPRT beads assay; The unit is calculated as 0.027 μmol per min per mg enzyme beads.

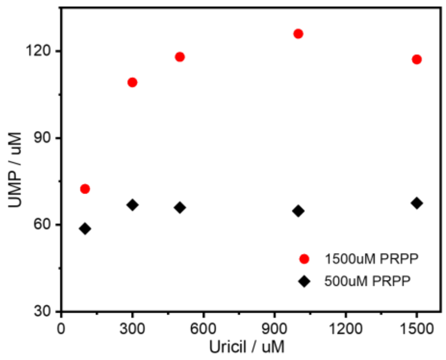

UPRT_beads: 8 ul **Flowrate**: 750 ul/h

PRPP: 500, 1500 uM

Uracil: 100, 300, 500, 1000, 1500 uM

Fig. S32. Activity characterization of UPRT beads in flow at steady state. Enzyme UPRT remains active after immobilization.

4.4.5 UMPK beads characterization

1 ml of assay mixture contains:

- IVTT Buffer
- 0,2 mM NADH (prepare stock freshly)
- 1.5 mM Phosphoenolpyruvate
- 1.5 mM UMP
- 3 mM ATP
- 2 U pyruvate kinase
- 2 U lactate dehydrogenase

  5 ul UMPK_beads

Calculate the activity of the *UMPK* beads solution according to:

$$U = \frac{(OD_{t2} - OD_{t1}) * 1000 * 0.5 * V_{reaction}}{(t2 - t1) * 6220 * V_{enzyme}}$$

Where t = elapsed time in minutes, OD is the linear absorbance change over $t_2 - t_1$, V_{enzyme} is the volume of enzyme solution added in µl and $V_{reaction}$ is the reaction volume in ml. Figure S33 and S34 show the activity calculation and characterization respectively.

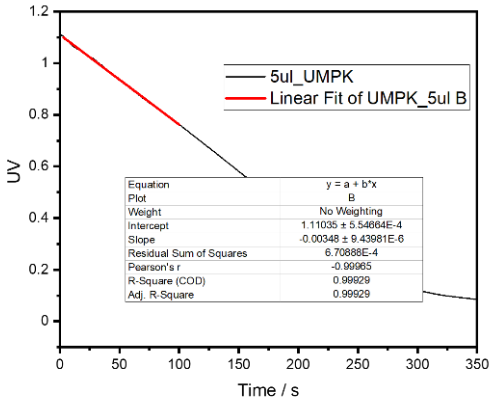

Fig. S33. Progress curve of UMPK beads assay; The unit is calculated as 0.1 µmol per min per mg enzyme beads.

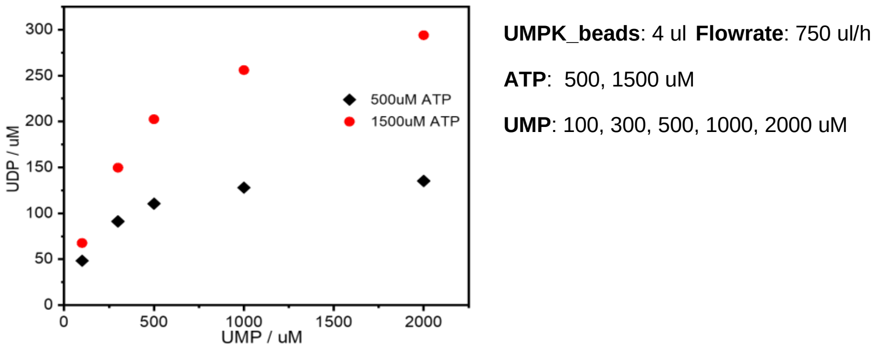

Fig. S34. Activity characterization of UMPK beads in flow at steady state. Enzyme UMPK remains active after immobilization.

4.4.6 GMPK beads characterization

1 ml of assay mixture contains:

- IVTT Buffer
- 0,2 mM NADH (prepare stock freshly)
- 1.5 mM Phosphoenolpyruvate
- 1.5 mM GMP
- 3 mM ATP
- 2 U pyruvate kinase
- 2 U lactate dehydrogenase

6 ul GMPK beads

Calculate the activity of the *GMPK* beads solution according to:

$$U = \frac{(OD_{t2} - OD_{t1}) * 1000 * 0.5 * V_{reaction}}{(t2 - t1) * 6220 * V_{enzyme}}$$

Where t = elapsed time in minutes, OD is the linear absorbance change over $t_2 - t_1$, V_{enzyme} is the volume of enzyme solution added in µl and $V_{reaction}$ is the reaction volume in ml. Figure S35 and S36 show the activity calculation and characterization respectively.

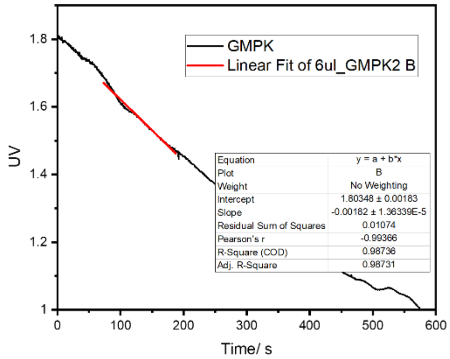

Fig. S35. progress curve of GMPK beads assay; The unit is calculated as 0.045 µmol per min per mg enzyme beads.

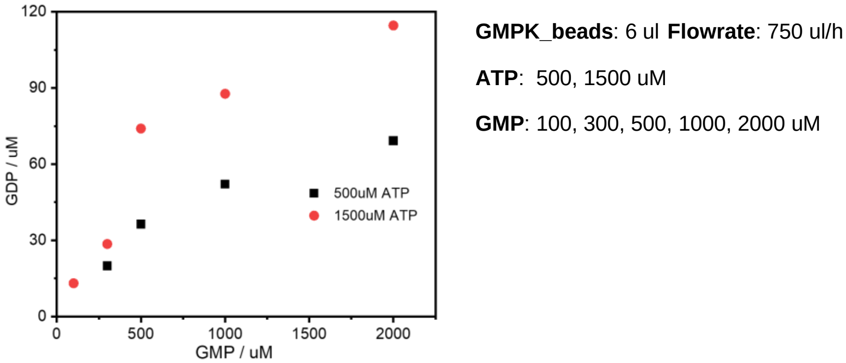

Fig. S36. Activity characterization of GMPK beads in flow at steady state. Enzyme GMPK remains active after immobilization.

4.4.7 Enzyme beads stability characterization

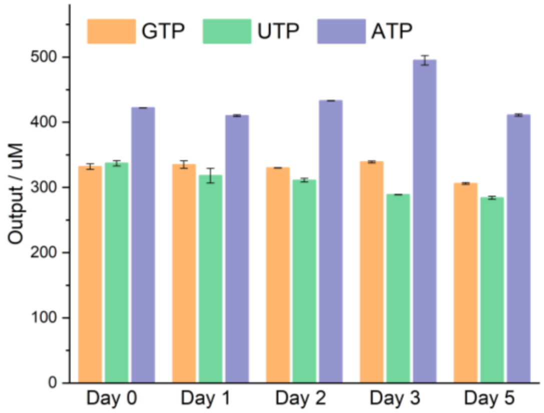

Fig. S37. Stability measurement in flow overtime; reaction conditions: Uracil, 496 uM; Adenine, 290uM; GMP, 484 uM; PRPP, 671 uM; PEP, 2169 uM; ATP 147 uM; flowrate144 ul/h; APRT 4ul; AK, 4ul; PK, 30ul; GMPK, 3.75ul; UPRT, 12 ul; UMPK, 33.75ul.

5. Supplementary References

1. Fröhlich, F., Weindl, D., Schalte, Y., Pathirana, D. *et al.* AMICI: high-performance sensitivity analysis for large ordinary differential equation models. *Bioinformatics* 37, 3676-3677 (2021).
2. Smith, L. P., Bergmann, F. T., Chandran, D. & Sauro, H. M. Antimony: a modular model definition language. *Bioinformatics* 25, 2452–2454 (2009).
3. Choi, K. et al. Tellurium: An Extensible Python-based Modeling Environment for Systems and Synthetic Biology. *Biosystems* 171, 74 (2018).
4. Hucka, M., Finney, A., Sauro, H. M., Bolouri, H., Doyle, J. C., et al. The systems biology markup language (SBML): A medium for representation and exchange of biochemical network models *Bioinformatics* 19 524–531 (2003).
5. Savitzky, A., & Golay, M. J. E., Smoothing and Differentiation of Data by Simplified Least Squares Procedures *Anal. Chem.* 36, 1627–39 (1964).
6. Yang, X. S., Nature inspired optimization algorithms ,Elsevier Science Publishers (2014).
7. Davis, L., Handbook Of Genetic Algorithms (1990).
8. Rosenbrock, H. H., An automatic method for finding the greatest or least value of a function *Comput J.* 3, 175–184. (1960).
9. Fister, J. R. I., Yang, X. S., Fister, I., Brest, J., & Fister, D., A Brief Review of Nature-Inspired Algorithms for Optimization *Electrotech. Rev.* 80. (2013).
10. Sörensen, K., Sevaux, M., & Glover, F., A History of Metaheuristics. (2017).
11. Sorensen, K., Metaheuristics-a metaphor exposed, IFORS (2015)
12. Mirjalili, S., & Lewis, A., The Whale Optimization Algorithm. *Adv. Eng.* 95, 51–67(2016).
13. Dorigo, M., Birattari, M., & Stutzle, T., Ant colony optimization. *IEEE Computational Intelligence Magazine*. 1, 28–39 (2006).
14. Kaveh, A., & Farhoudi, N., A new optimization method: Dolphin echolocation. *Adv. Eng.* 59, 53–70 (2013).
15. Krishnanand, K. N., & Ghose, D., Detection of multiple source locations using a glowworm metaphor with applications to collective robotics. *Proceedings 2005 IEEE Swarm Intelligence Symposium*.
16. Deb, S., Fong, S., & Tian, Z. H., Elephant Search Algorithm for optimization problems. *2015 Tenth International Conference on Digital Information Management (ICDIM)* 249–255.
17. Duman, E., Uysal, M., & Alkaya, A. F., Migrating Birds Optimization: A new metaheuristic approach and its performance on quadratic assignment problem. *Inf.* 217, 65–77, (2012).
18. Atashpaz-Gargari, E., & Lucas, C., Imperialist competitive algorithm: An algorithm for optimization inspired by imperialistic competition". *2007 IEEE Congress on Evolutionary Computation* 4661–4667.
19. He, Y. C., & Wang, X. Z., Group theory-based optimization algorithm for solving knapsack problems, *Knowledge-Based Systems* 219, (2021).
20. Raue, A., et al. Structural and practical identifiability analysis of partially observed dynamical models by exploiting the profile likelihood. *Bioinformatics* 25, 1923–1929 (2009).
21. Brun, R., Reichert, P. & Künsch, H. R., Practical identifiability analysis of large environmental simulation models. *Water Resources Research* 37, 1015–1030 (2001).
22. Aguiar, P. F., Bourguignon, B., Khots, M. S., Massart, D. L., & Phan-Than-Luu, R., D-optimal designs. *Chemometr Intell Lab Syst* 30, 199-210 (1995).
23. Gábor, A., Villaverde, A. F. & Banga, J. R. Parameter identifiability analysis and visualization in large-scale kinetic models of biosystems. *BMC Systems Biology* 11, 1–16 (2017)
24. Ruess, J., Andreas, M.-A., & John. L., Designing experiments to understand the variability in biochemical reaction networks *J. R. Soc. interface.* 10, 20130588 (2013)
25. Smith, Robert W., van Sluijs, B., & Fleck. C., Designing synthetic networks in silico: a generalised evolutionary algorithm approach.*BMC Systems Biology* 11, 1-19 (2017).

26. van Sluijs, Bob, et al. A microfluidic optimal experimental design platform for forward design of cell-free genetic networks. *Nat. Commun.* 13, 3626 (2022).
27. Cook, P.,F., & Cleland. W. W., Enzyme kinetics and mechanism Garland Science, 2007.
28. Dobson, G. P., Hitchins, S., & Teague, W. E., Thermodynamics of the pyruvate kinase reaction and the reversal of glycolysis in heart and skeletal muscle. *J. Biol. Chem.* 277, 27176-27182 (2002).
29. Israelsen, W. J., & Heiden, M. G. V., Pyruvate kinase: Function, regulation and role in cancer. *Semin Cell Dev Biol.* 43, 43-51 (2005).
30. Rohwer, J. M., Arno J. H., & Jan-Hendrik S. H., A universal rate equation for systems biology. ESCEC 2006.
31. Baltussen, M., Wiel, J., Regueiro, C., Jakštaitė M., & Huck, W. T. S., A Bayesian approach to extracting kinetic information from artificial enzymatic networks, *Anal. Chem.* 94, 7311 (2022).
